# The effect of S-substitution at the O6-guanine site on the structure and dynamics of a DNA oligomer containing a G:T mismatch

**DOI:** 10.1371/journal.pone.0184801

**Published:** 2017-09-14

**Authors:** Elaine Ann Moore, Yao-Zhong Xu

**Affiliations:** School of Life, Health and Chemical Sciences, STEM Faculty, The Open University, Milton Keynes, Buckinghamshire, United Kingdom; Institut Pasteur, FRANCE

## Abstract

The effect of S-substitution on the O^6^ guanine site of a 13-mer DNA duplex containing a G:T mismatch is studied using molecular dynamics. The structure, dynamic evolution and hydration of the S-substituted duplex are compared with those of a normal duplex, a duplex with S-substitution on guanine, but no mismatch and a duplex with just a G:T mismatch. The S-substituted mismatch leads to cell death rather than repair. One suggestion is that the G:T mismatch recognition protein recognises the S-substituted mismatch (G^S^:T) as G:T. This leads to a cycle of futile repair ending in DNA breakage and cell death. We find that some structural features of the helix are similar for the duplex with the G:T mismatch and that with the S-substituted mismatch, but differ from the normal duplex, notably the helical twist. These differences arise from the change in the hydrogen-bonding pattern of the base pair. However a marked feature of the S-substituted G:T mismatch duplex is a very large opening. This showed considerable variability. It is suggested that this enlarged opening would lend support to an alternative model of cell death in which the mismatch protein attaches to thioguanine and activates downstream damage-response pathways. Attack on the sulphur by reactive oxygen species, also leading to cell death, would also be aided by the large, variable opening.

## Introduction

**Fig 1:**
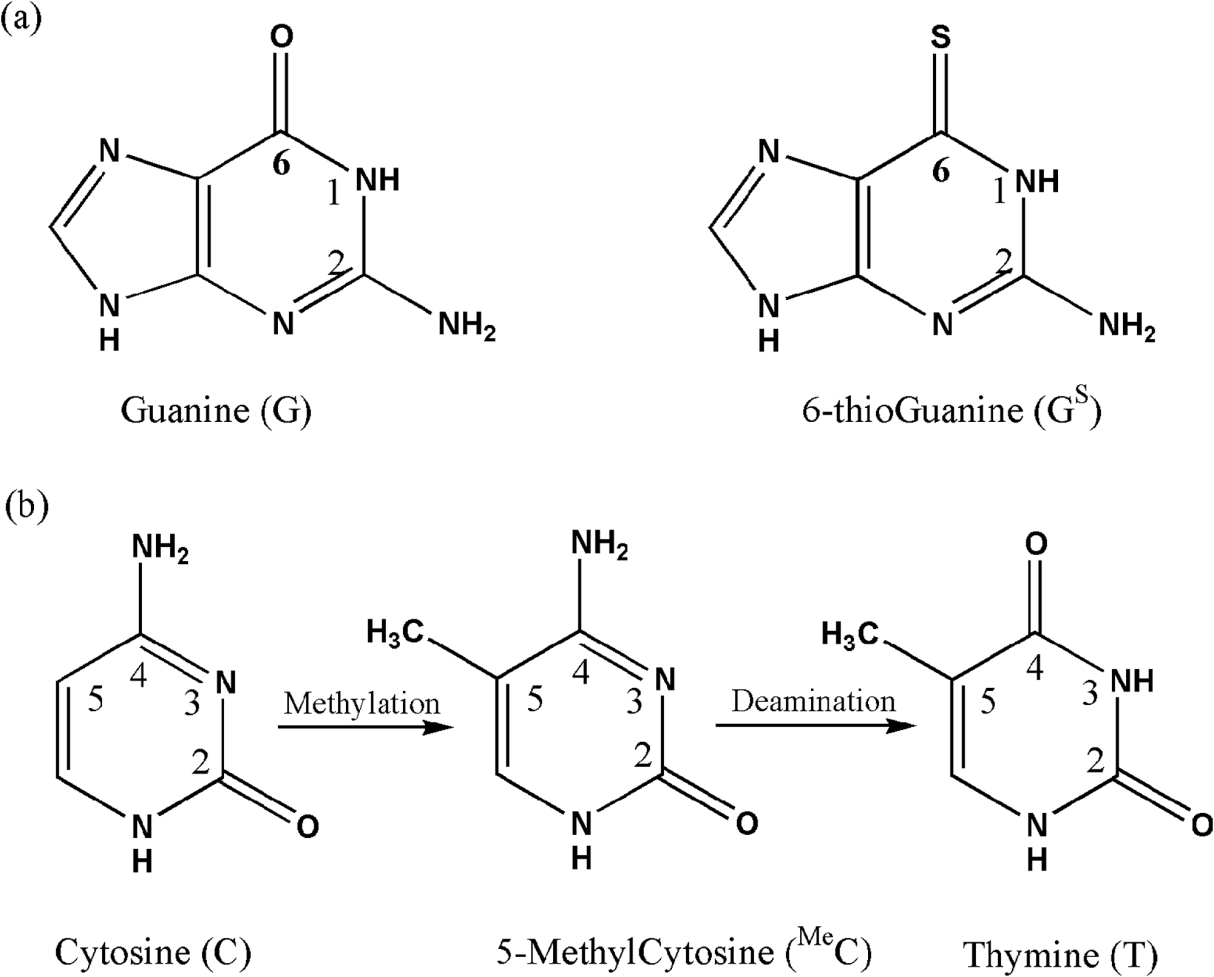
(a) Chemical structures and labelling of guanine and 6-thioguanine, (b) the conversion of cytosine to thymine.

6-thioguanine, (Fig 1a), is an important drug against childhood leukaemia and its effects are thought to be due to its incorporation into DNA [1]. Although the incorporation of 6-thioguanine (which we shall designate G^S^) does not lead to disruption of the overall structure of DNA, there is evidence that it renders one of the DNA repair mechanisms less efficient. Cytosine is readily methylated to form 5-methylcytosine, ^Me^C. The 4-amino group of 5-methylcytosine of the G: ^Me^C pair is deaminated to form thymine (see Fig 1b) resulting in a G:T pair. Such a mismatch would lead to a G:C to A:T mutation and hence alteration of the code with possibly deleterious biological effects. Yuan et al [2] have shown that such a mutation also occurs with G^S^:C.

The recognition of genetic errors (such as G:T mismatches) is an initial and crucial step in the repair of damaged DNA. Therefore species must possess effective repairing systems. In *E coli* a protein complex, the MutS/MutL/MutH mismatch repair system, will repair G:T mismatches. The human equivalent (MutSalpha/MutLalpha) will also carry out this maintenance. The mismatch repair system will recognise G^S^:T to a similar extent as G:T implying that the human system can repair G^S^:T [3]. However this repair replaces T by C thus restoring the original G^S^:C pair. One model suggests that this cycle of futile repair eventually leads to DNA breakage and cell death [4,5]. This means that probably only a small proportion of the incorporated G^S^ may be removed by the mismatch repair system. An alternative model suggests the mismatch repair system proteins will bind to G^S^ but instead of repairing the G^S^:T mismatch will activate damage-response pathways downstream leading to apoptosis [6, 7].

The recognition and repair of G:T mismatches has been extensively studied experimentally [8]. In the recognition step, MutS or MutSalpha is thought to travel along the DNA strand testing the base pairs. The exact way in which the G:T mismatch is recognised is still not entirely clear, but it has been suggested that the protein acts to test if the base pair will readily adopt a particular conformation. In the X-ray co-crystal structure of MutS bound to a G:T mismatch-containing DNA oligomer, the DNA substrate is significantly deformed, including an approximately 60° bend into the major groove [9]. The bend is accompanied by insertion of a MutS side chain, Phe36, into the helix. However the crystal structure conformation is not necessarily that adopted *in vivo*. Tessmer et al [10] suggest that the Phe residue promotes the unbending, not bending, of DNA at mismatch sites and that formation of the specific unbent MutS-DNA conformation at mismatches appears to be required for the inhibition of ATP hydrolysis by MutS that signals initiation of repair.

To complement the experimental results, we have investigated structural features using calculations. To approach the conditions found *in vivo*, it is necessary to consider the behaviour of mismatched DNA base pairs in aqueous solution and close to physiological conditions. In such conditions, DNA can sample a large number of conformations. A molecular dynamics approach which follows changes in conformations is therefore preferable for such a study to an approach that only considers the lowest energy conformation. To provide a good model for our study a short double strand of DNA is selected, sufficiently long that we can replace bases in the middle of the strand without significantly affecting the end-bases. Because of the large number of atoms in duplexes, ab initio studies are not feasible and therfore molecular dynamics with molecular mechanics using parameters designed for DNA are generally used. This also enables the study of the fluctuations in conformation as a function of time. Studies using NMR spectroscopy and computer modelling [11, 12] show that oligomers containing G:T mismatches adopt a B-DNA structure without major structural changes of the backbone conformation. However there are significant local structural changes at the mismatch site. In particular it was noted that the variation in major groove width was greater for the mismatched duplex. This would support an earlier suggestion that the difference in local flexibility at the mismatch site is important for mismatch recognition [13].

**Fig 2:**
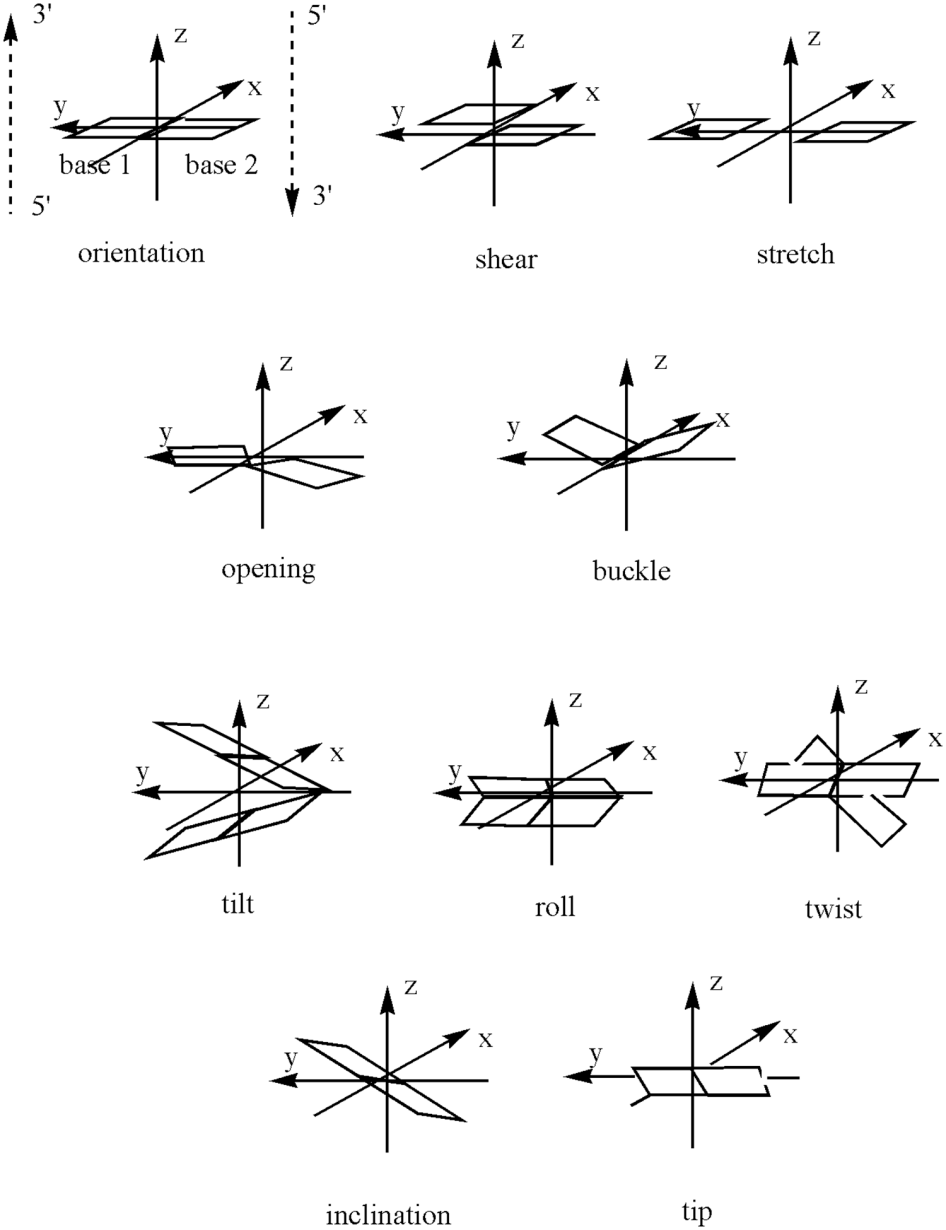
Parameters describing duplex structure [16].

Structural and dynamic effects of replacement of the O6 of guanine by S6 on the base G7 in the duplex d(5’-GCTAAG**X**AAAGCC-3’, X = G or G^S^) have been studied by Somerville et al [14] using molecular dynamics over 250 ps with NMR restraints. They found that the G^S^-substituted duplex was less stable than the normal duplex but that there were only modest perturbations to the DNA structure. They did note however an approximately 10° opening (see Fig 2) of the mismatch base pair towards the major groove. NMR data showed that the G^S^7 imino proton exchanged more rapidly with water than the G7 imino proton. Proton exchange experiments also indicated that the base pair lifetime was reduced. Šponer et al [15] studied the effect of G^S^ for G substitution in G-rich duplexes, triplexes and tetraplexes found that substitution of guanine by 6-thioguanine was found to destabilise the structure and to shift the geometry from the B/A intermediate form to the pure B-form. No visible perturbation of the duplex structure was observed but for a single G→G^S^ mutation, changes at the mutation site in the helical twist, roll, negative slide and inclination were noted. Changes in hydration pattern were also noted with poorer hydration of S6 relative to O6. Šponer et al also reported calculations on Somerville’s duplex and confirmed that the presence of G^S^ did not introduce major alterations to the structure. Their simulations did not, however, support the existence of anomalous helical parameters as suggested by Somerville’s data.

Bohon and de los Santos [17] have considered an 11-base oligomer duplex containing G^S^ both in the normal form (G^S^:C) and with a mismatch at the 6-thioguanine site (G^S^:T). This work concluded surprisingly that the oligomer duplex containing the G^S^:T mismatch was more stable than the one containing G^S^:C. *In vacuo* molecular dynamics with restraints from NMR spectroscopy gave a structure in which G^S^:T assumed a wobble-type base pairing. A base-pair buckle (Fig 2) of -11° was noted. As with G:T mismatch and G^S^:C pairings, the disruption from G^S^:T to the structure was localized.

In this paper, we compare the effect of a C → T mutation on a 13-base oligomer duplex d(5’-GCTAA**X**GAAAGCC-3), where X = G or G^S^, using molecular dynamics. Following Šponer et al [15] we performed the calculations with explicit water, that is with the duplexes surrounded by a large number of water molecules each of whose interaction with the duplex and each other is calculated explicitly.

## Methods

The results discussed in this paper are based on molecular dynamics trajectories for double-stranded B-DNA oligomers, each containing 13 base pairs. The sequences were based on d(5’-GCTAA**G**GAAAGCC-3’). As well as the duplex with i) normal G:C base-pairing, we also ran oligomers with ii) a G:T mismatch, iii) G^S^:C and iv) a G^S^:T mismatch at the G6 site (see Table 1).

**Table 1:** DNA sequences used in this study.

	normal	G:T mismatch	G^S^:C	G^S^:T mismatch
**X**	G	G	G^S^	G^S^
**Y**	C	T	C	T

5’-GCTAA**X**GAAAGCC-3’3’-CGATT**Y**CTTTCGG-5’

Starting structures were constructed in the standard B-like conformation using the Nucgen module of AMBER10 [18]. The force field used was the parmbsc0 modification [19] to the parm99 force field [20, 21]. Parameters for 6-thioguanine (G^S^) were based on those given by Šponer et al [15] but with the atomic charges recalculated using RESP methodology to match the parmbsc0 parameters. (see Supplementary Data S1 Table).

Simulations were carried out using the AMBER10 suite of programs. Sodium ions were added to ensure a zero net charge for the solute-counterion complex and the system hydrated with a truncated octahedral box of TIP3P [22] water molecules forming a 1.2 nm buffer around the duplex. This gave us 7280 water molecules for the unmodified oligomer, 7178 for the oligomer with a G:T mismatch, 7505 for the G^S^:C oligomer and 7195 for the G^S^:T mismatch. Water molecules play an important role in nucleic acid structures and this allows us to look at the interaction of the oligomers with surrounding water molecules. In vacuo calculations on duplexes containing normal bases can lead to unravelling of the duplex. Initial equilibration, involving energy minimization of the solvent, then of the solute–solvent system was followed by thermalization of the solvent at constant volume over 20 ps. For the duplexes containing 6-thioguanine an initial stage of energy minimization of the duplex was included. Unrestrained production simulations over 15 ns were carried out at constant pressure using the Langevin temperature equilibration scheme [23] with a collision frequency of 1.0 ps^-1^. This scheme has been shown to give accurate structures without requiring restraints. All simulations were carried out using periodic boundary conditions and the particle mesh Ewald method [24]. All chemical bonds involving hydrogen atoms were restrained using SHAKE [25], allowing for stable simulations with a 2 fs time step. Conformational snapshots were saved every 40 ps initially and then every 80 ps. The resulting conformations were grouped into clusters of similar rms of the average distance to the centroid of the duplex.

The results were analysed using the ptraj module in AmberTools. In addition conformational analysis on structures averaged over each 1 ns of calculations and on the average structure of the most-visited cluster of structures was performed using 3DNA [16], which provides a full set of helical, backbone and groove geometry parameters. These are defined in Fig 2. The ptraj module of AmberTools was used to obtain distances between the potentially hydrogen-bonded atoms as a function of time.

## Results and discussion

Plots of the rms deviation of the backbone are given in the Supplementary Data (S1–S4 Figs). There are no major deviations for any of the four oligomers.

First we consider the effect of the G:T mismatch on the duplex structure and then the effect of G^S^. Finally we will consider the effect of both a mismatch and the replacement of G by G^S^.

### G:T duplex

**Fig 3:**
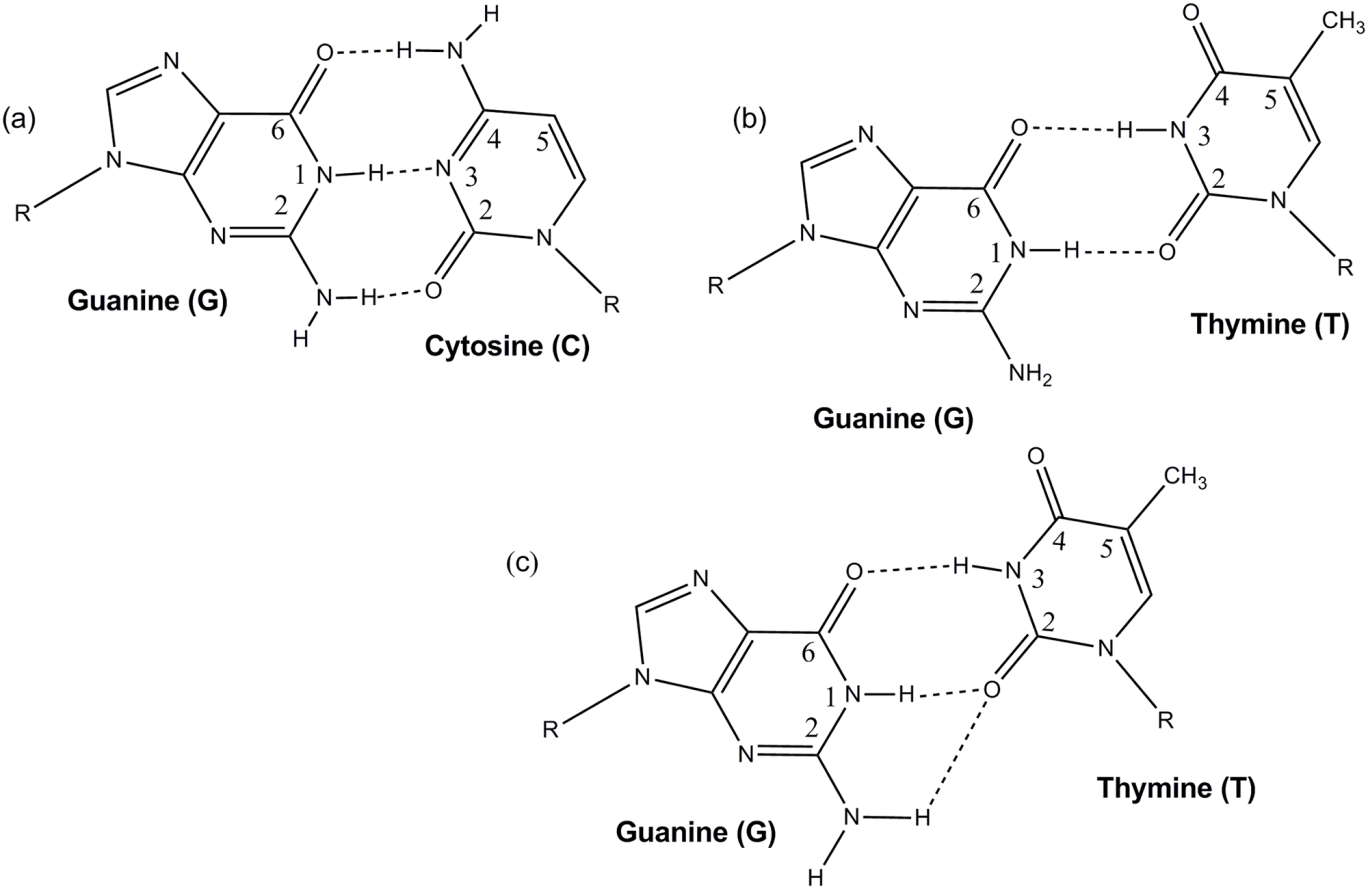
Structures of (a) paired G:C (b) paired G:T with wobble base pair structure and (c) paired G:T with bifurcated structure.

The structure and dynamics of the G:T mismatched oligomer is important as it is differences in these properties from those of the normal oligomer that are likely to be detected by the repair mechanism. At the level of the individual bases there will be differences due to the change in hydrogen-bonding between the paired bases. In the normal oligomer, G and C are linked by three hydrogen bonds between O6 on G and N4 on C, N2 on G and O2 on C and N1 on G and N3 on C (see Fig 3a). When C is replaced by T, O6 on G now hydrogen-bonds to N3 on T and N1 on G hydrogen-bonds to O2 on T leaving N2 on G without an obvious atom to form a hydrogen bond with (see Fig 3b). The structure without a bond from N2 on G to T is known as the wobble base pair structure. An alternative is for both N1 and N2 to hydrogen bond to O2 on T–the bifurcated structure (Fig 3c) [12]. The G:T mismatch duplex adopted the wobble base pair structure (Fig 3b) over the majority of the trajectory. and on average the most-visited structures identified by 3DNA adopted this form.

**Fig 4:**
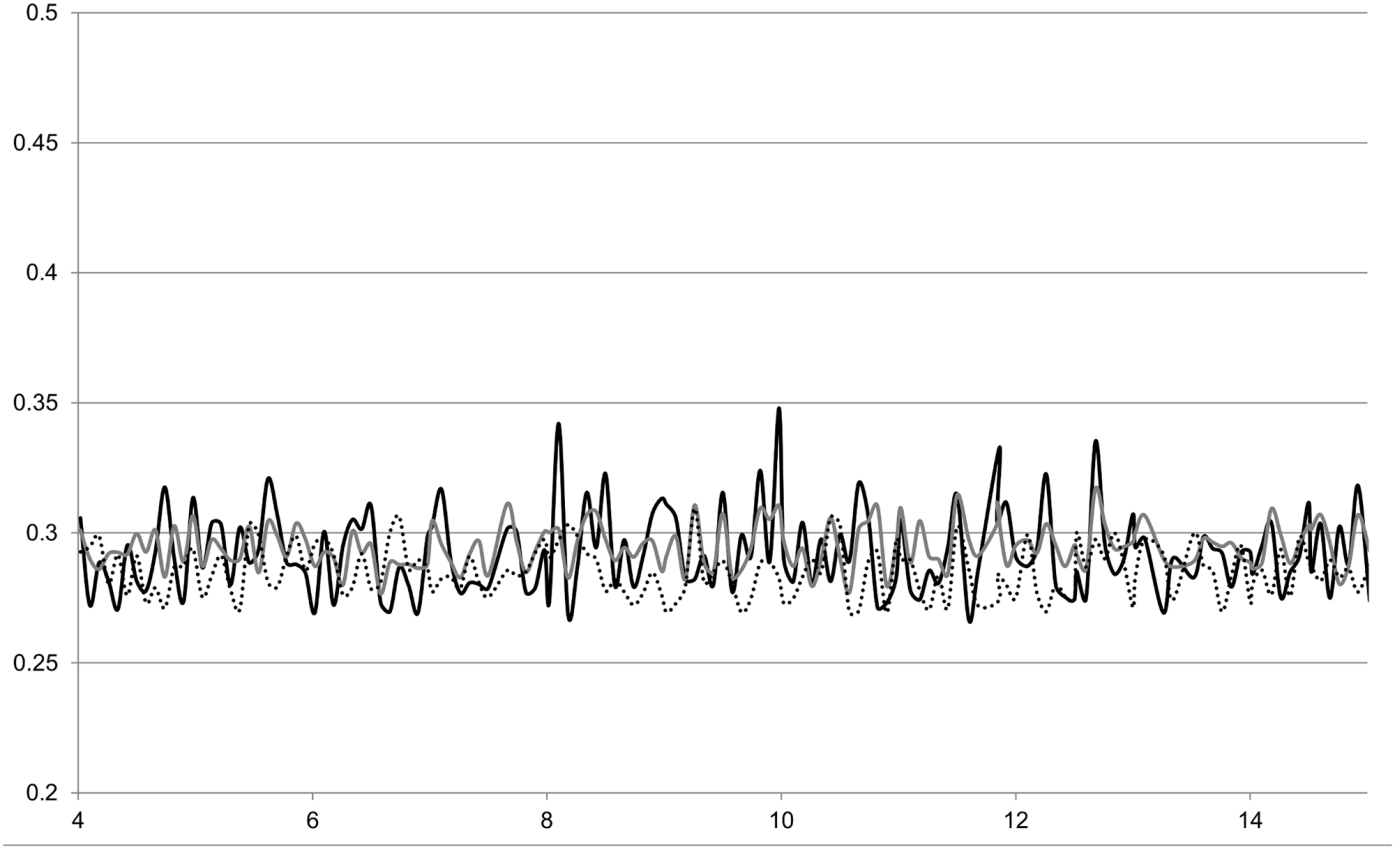
Variation of distances between potentially hydrogen-bonded atoms with time for the G:C duplex. Black line O6-N4 distance, grey line N1-N3 distance, dotted line N2-O2 distance.

**Fig 5:**
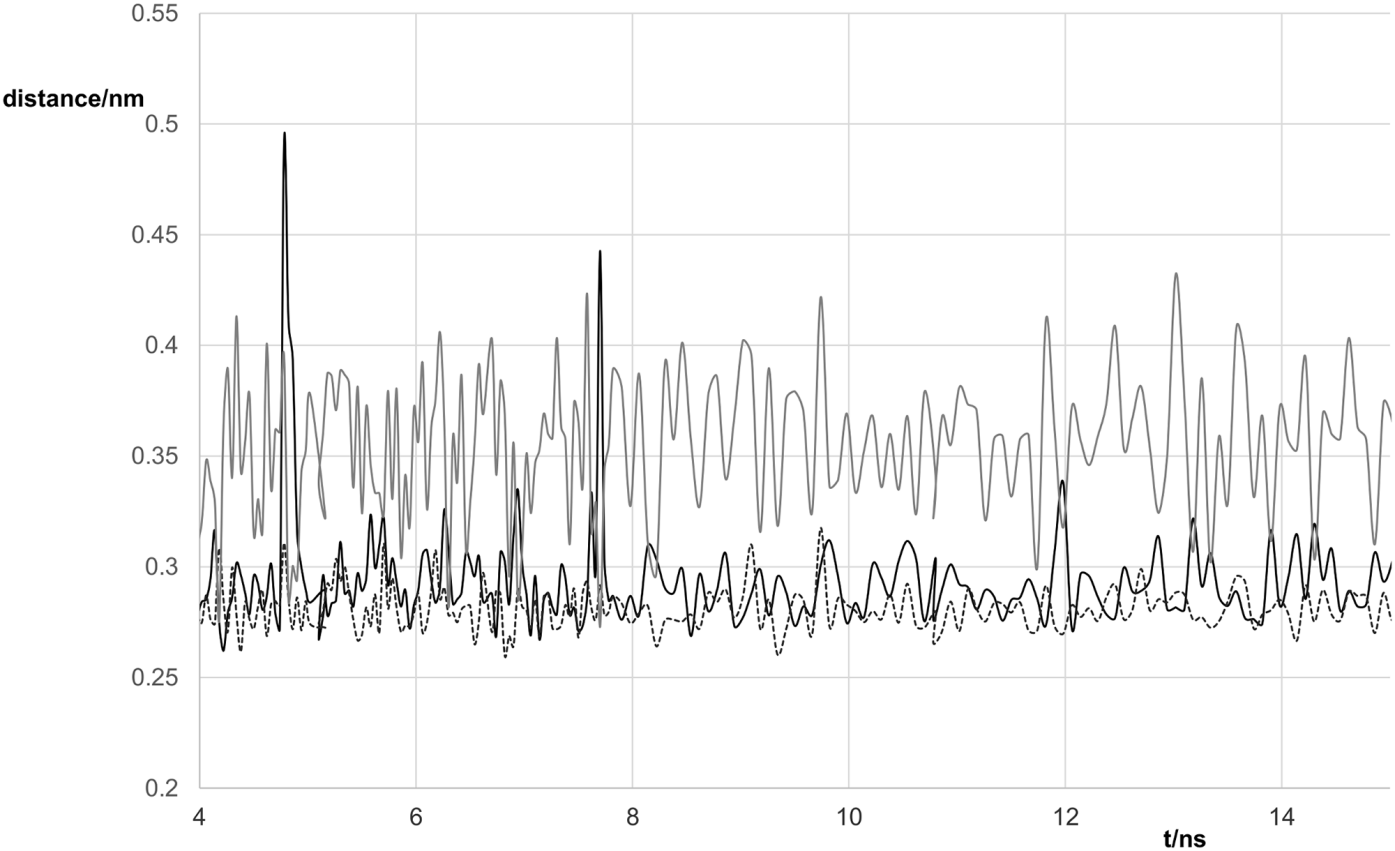
Variation of distances between potentially hydrogen-bonded atoms with time for the G:T duplex. Black line O6-N3 distance, grey line N2-O2 distance, dashed line N1-O2 distance.

The distances between the potentially hydrogen-bonded atoms as a function of time are plotted in Figs 4 and 5. The O6-N3 and N1-O2 hydrogen bonds give the wobble base pair structure. The bifurcated structure has N1-O2 and N2-O2 hydrogen bonds.

For the G:C oligomer, the O6-N4, N2-O2 and N1-N3 distances remained close enough to hydrogen bond throughout and with donor–H–acceptor angles showing less than 20° deviation from a straight line. For the G:T oligomer, the N1-O2 distance remains short enough for hydrogen-bonding throughout. The O6-N3 distance is generally short enough to indicate hydrogen-bonding but occasionally increases to a value too large for efficient hydrogen-bonding. These two hydrogen-bonds are those contributing to the wobble base pair structure. Again the donor–H–acceptor angle deviated by less than 20° from a straight line. The N2-O2 hydrogen-bond is involved in the bifurcated structure identified by Alvarez-Salgado et al [12]. The N2-O2 distance is generally rather long for hydrogen-bonding but it occasionally dips to a lower value. Analysis of possible hydrogen-bonding pairs in the case of G:T using ptraj showed that the N1-O2 atoms remained within 0.35 nm of each other all the time (dashed line in Fig 5). However, although the O6-N3 distance (black line in Fig 5) was less than 0.35 nm 98% of the time, the lifetime of this hydrgogen-bond was markedly reduced relative to that of the N1-O2 hydrogen-bond. Analysis of the N2-O2 distance (grey continuous line in Fig 5) using the hydrogen-bond option of ptraj, indicated that these atoms were within 0.35 nm about 40% of the time but the N2-H-O2 angle was too far from 180° to form a strong hydrogen-bond. This suggests that the wobble base pair structure is the dominant form.

The mismatch recognition protein detects a change in the shape of the section of the DNA helix where the mismatch lies [10]. So what effect does the difference in local base pair hydrogen bonding have on the structure of the oligomer?

As an example, differences between the average conformation of the most-visited cluster G:C and G:T oligomers are summarised in Table 2. These values are typical of those found in the analysis of the conformations averaged over 1 ns. Data on the average values for individual nanoseconds can be found in the Supplementary Data (Tables A, B, C, D in S1 Text).

**Table 2:** Structural parameters of the average structures of the most-visited clusters of the normal (G:C) and mismatched (G:T) duplexes at the G:X site.

Property*	Normal duplex	Mismatched duplex
**Major groove/nm**	2.16	1.84
**Shear/nm**	-0.25	-2.27
**Stretch/nm**	-0.11	-0.13
**Opening/°**	0.42	0.65

* Some of the properties are illustrated in Fig 2

**Fig 6:**
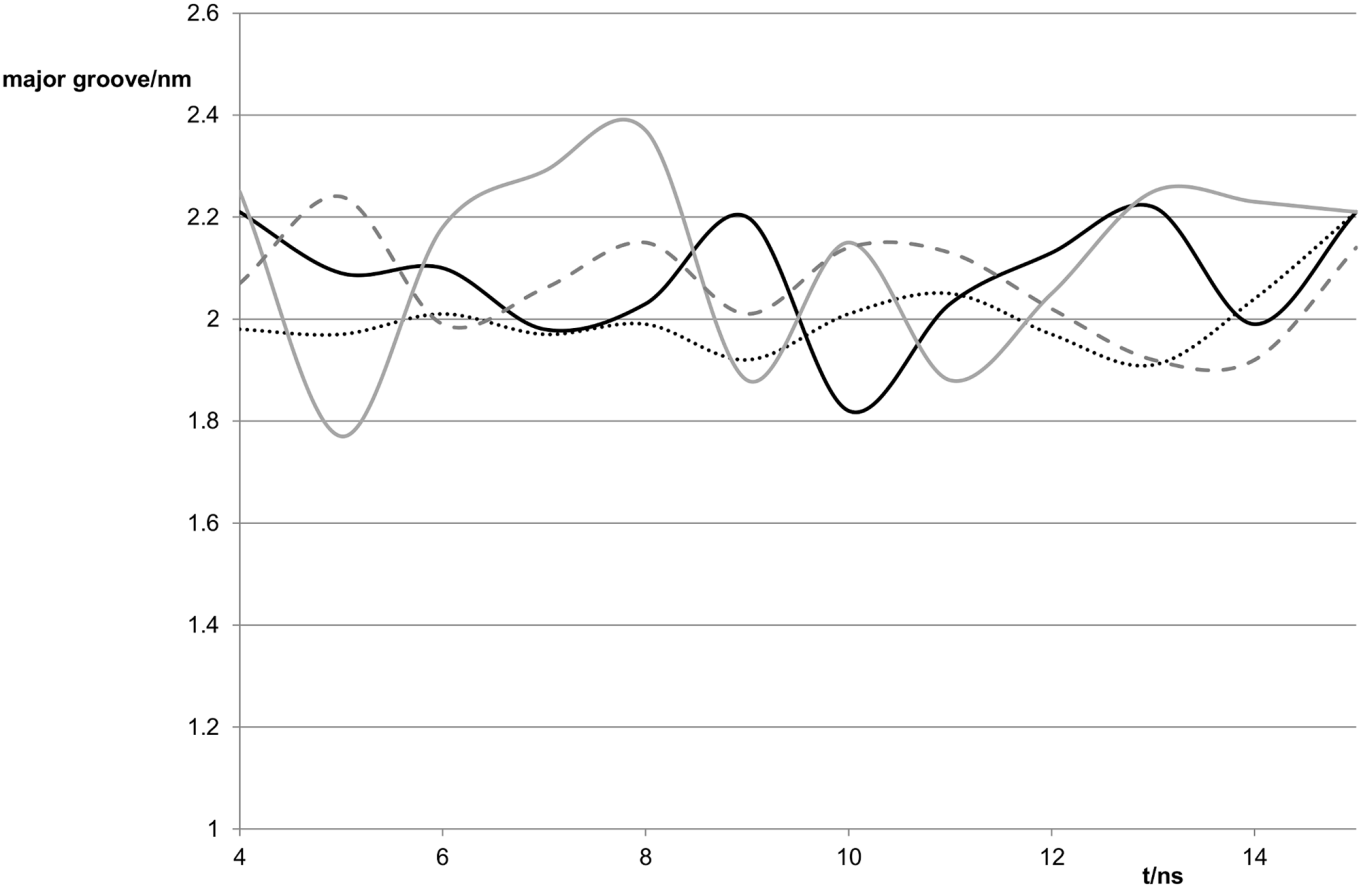
Variation of the width of the major groove with time for the four duplexes studied. Continuous black line G:C duplex, dotted line G:T duplex, continuous grey line G^S^:C duplex and dashed line G^S^:T duplex.

The major groove for the mismatch duplex at the G:T site was about 0.3 nm less than that of the normal G:C duplex. Shear, stretch and opening are characteristic of a particular base pair [16]. Table 2 shows a distinct difference in the shear values. Looking at the values of the parameters over time gives an insight as to their variability. A measure of the major groove width associated with a particular dinucleotide step, n, can be obtained from the distance between the P atom of the phosphate group on base n-2 on one strand where the bases are numbered in the 5’ - 3’ direction to the P atom of the phosphate group on base n+2 in the other strand where the numbering is in the 3’– 5’ direction [26]. An analysis of such P-P distances found that the rmsd of the unrefined major groove for individual time steps at the G:T site for the mismatch duplex was lower than for the G:C duplex, thus suggesting that there is greater variability for the G:C duplex on a 80 ps timescale. By plotting the averages for each nanosecond in Fig 6, we can see that there is also less variation in the refined major groove for the G:T mismatch than for the normal G:C duplex on the nanosecond timescale.

Our data agree with the result stated in [11] that the minor grooves of the normal and G:T mismatch duplexes are similar in both average value and range of values.

Inspection of the base pair step parameters shows differences in the roll and twist with the twist being nearly 10° larger for the G:T mismatch. In addition the inclination of the average structure of the most-visited cluster of the G:T duplex was over 15° lower than that of the G:C duplex. Tip was about 4° higher and helical twist nearly 9° higher. These two parameters also showed less variation at the nanosecond time scale than those for the G:C duplex.

It is known that the conformation of a base pair is influenced by the nature of the neighbouring base pairs, for example Lavery et al [27] show that nearest-neighbour effects on base pair steps are very significant. This has two consequences 1) the changes we obtain at the mismatch site may vary from previous work in which the nearest-neighbour base pairs differed and 2) there may be significant changes in the parameters of adjacent base pairs. Inspection of the base pair step parameters of the average of the most-visited cluster of G:T mismatch duplex shows that although the average twist for all the steps (33.23°) is similar to that of the normal duplex (31.50°) the standard deviation (4.03 compared to 2.17) is greater. The main cause of this deviation is that the value for the AG/TT step is much smaller than that of the AG/CT step in the normal oligomer and the value for the GG/CT is much larger than that of the GG/CC step as shown in Table 3

**Table 3:** Base pair step parameters for the average structure of the most visited cluster of the four duplexes studied.

step	Shift/nm	Slide/nm	Rise/nm	tilt/°	roll/°	twist/°
**GG/CC**	-0.007	-0.169	0.366	1.83	7.77	29.47
**GG/CT**	-0.024	-0.134	0.356	-0.51	-0.95	39.11
**G**^**S**^**G/CC**	-0.028	-0.093	0.359	2.28	2.75	31.30
**G**^**S**^**G/CT**	-0.144	-0.054	0.354	-0.15	2.51	40.58
**AG/CT**	0.010	-0.127	0.324	-1.70	4.08	28.58
**AG/TT**	0.064	-0.185	0.344	-1.87	4.49	21.91
**AG**^**S**^**/CT**	0.048	-0.092	0.337	-1.15	1.00	32.57
**AG**^**S**^**/TT**	0.92	-1.51	0.343	-3.75	3.37	24.05

The mismatch recognition is thought to depend on structural features [8–10, 13], thus candidates would be the switch from the three hydrogen-bonds holding G and C together to the wobble base pair of G:T giving rise to the differences we have noted in Tables 2 and 3. Overall the oligomer containing G:T had a less variable structure and it could be suggested that this lack of variability, especially in the major groove, would enable the mismatch protein to attach more easily. We note however that previous work [11] on another G:T-containing duplex showed greater variability in the width of the major groove than for the normal duplex. Since the mismatch recognition mechanism must be the same for all strands of human DNA containing the G:T mismatch, this suggests that the average structure rather than the variability is recognised.

As the recognition takes place in an aqueous environment, the possibility of water playing a role should be considered. One way of doing this is to compare the average number of water molecules within a particular distance of atoms in the base pairs over the time modelled in the calculations. Those within 0.34 nm constitute the first shell and those between 0.34 nm and 0.50 nm form the second shell. The numbers of water molecules in the first and second shells surrounding G6 were very similar for G:C and G:T, on average 46 in the first shell and 105 for G:C and 103 for G:T in the second shell. The number in the first shell for both bases is similar to that found for water molecules close to the major groove in guanine, cytosine, adenine and thymine by X-ray crystallography [28]. The H-bond option of ptraj was also used to investigate the interaction of O6 on guanine with solvent molecules. Water molecules were found with the oxygen within 0.35 nm of O6 on guanine for both G:C and G:T. The frequency of this geometry was however low 7.65% for G:C and 3.65% for G:T In addition G:C had solvent molecules close to N2 on guanine,. This was not found for G:T. The donor-H-acceptor angles deviated however by 20–30° from 180°, suggesting that these were weakly if at all hydrogen-bonded.

### G^S^:C duplex

**Fig 7:**
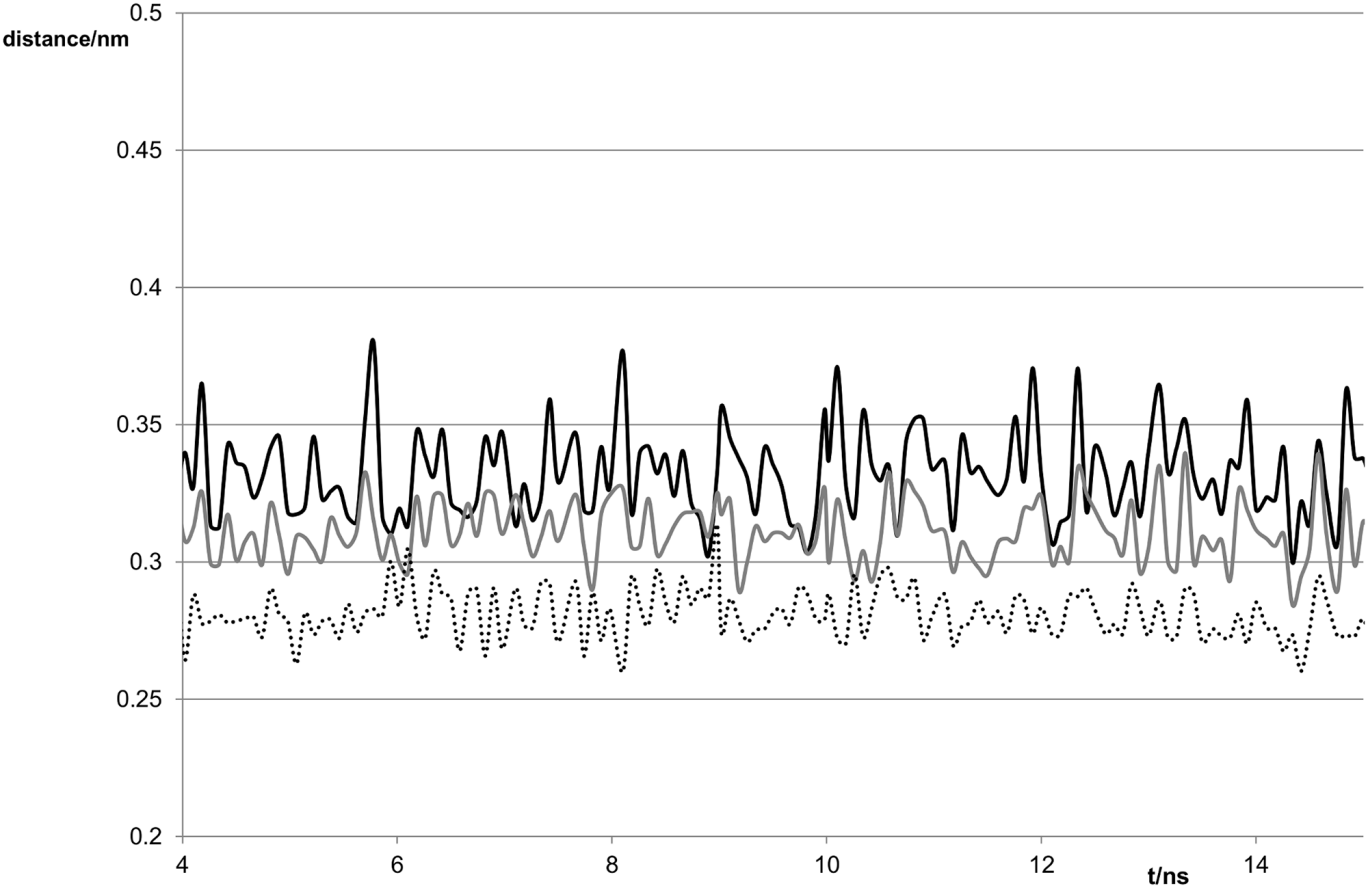
Variation of distances between potentially hydrogen-bonded atoms with time for the G^S^:C duplex. Black line S6-N4 distance, grey line N1-N3 distance, dotted line N2-O2 distance.

In our calculations the three hydrogen bonds linking G^S^ and C in the duplex, (S6 on G and N4 on C, N2 on G and O2 on C, and N1 on G and N3 on C) persisted over the entire time of the simulation (see Fig 7). The S-N distance is longer than the O-N distance as expected and the values are similar to those obtained by ab initio calculations on the base pairs [29].Values of the major and minor grooves were similar to those of the G:C duplex, in contrast to the change in the width of the major groove observed for the G:T mismatch.

Local base pair parameters at the G^S^:C site were generally similar to those for the G:C duplex with the exception of opening which was about 7° larger, see Table 4. The shear and stretch values resembled those of the G:C oligomer rather than those of the G:T oligomer.

**Table 4:** Structural parameters of the average structures of the most-visited clusters of the G^S^:C and G^S^:T duplexes at the G:X site.

Property*	G^S^:C	G^S^;T
**Major groove/nm**	2.08	1.98
**Shear/nm**	0.002	-0.216
**Stretch/nm**	0.013	-0.012
**Opening/°**	7.18	18.56
**Helical twist/°**	31.5	40.65

* Some of the properties are illustrated in Fig 2

**Fig 8:**
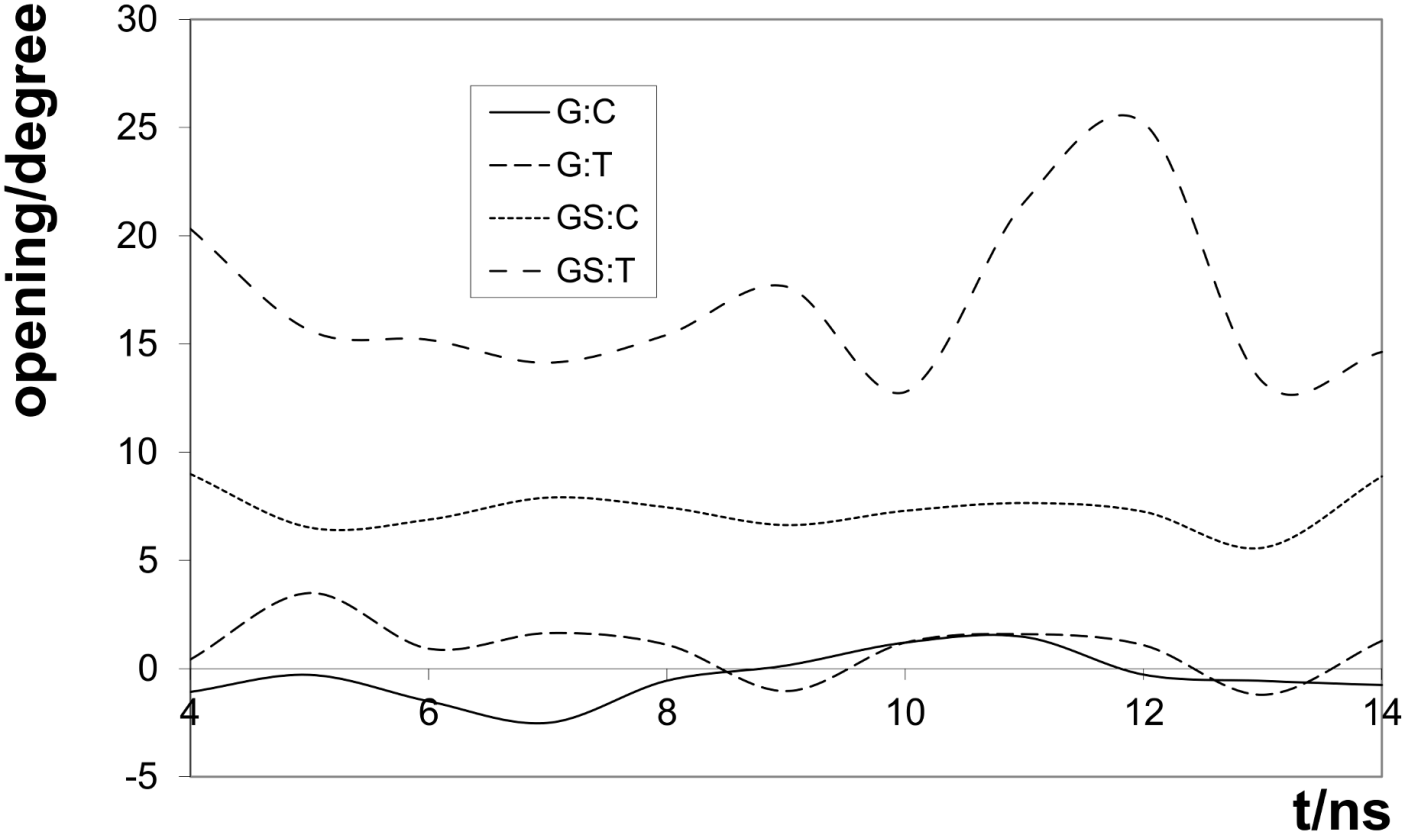
Comparison of opening for S-substituted and non-substituted duplexes.

Fig 8 shows the persistence of an increase in opening for G^S^-containing duplexes. This is not unexpected as the S6-N4 hydrogen-bond would be expected to be longer than the O6-N4 hydrogen-bond of the G:C duplex.

Because the sulfur atom is larger than oxygen atom and less electronegative, the interaction of water with thioguanine is expected to differ from that with guanine. Ab initio calculations on the interaction of the isolated uracil and thiouracil with water molecules [30] find that the binding energies of uracil and thiouracil with one water do not vary markedly, but calculations with three water molecules suggest that the first hydration shell differs. However in the duplexes O6 and S6 are hydrogen-bonded to a base in the other strand and thus less available to form the interactions with water found for the isolated bases. Further as substitution of guanine by thioguanine is not recognised by the mismatch repair mechanism, any differences between the hydration of the G:C duplex and G^S^:C duplex would not be expected to play a role in the recognition process. The average number of water molecules in the first and second shells around G^S^6 were similar to that for the G:C duplex, 46 and 103 respectively. Water molecules were found with oxygen within 0.35 nm of S6 on thioguanine 0.61% of the time for G^S^:C. Solvent interactions with N2 on thioguanine, and O2 and N4 on cytosine were not recorded. This agrees with the conclusion of Šponer et al [15] that S6 is less hydrated than O6.

Roll was generally lower for the G^S^-containing duplex than for the normal oligomer, in agreement with the previous work [12], but other local base pair step parameters were broadly similar (see Table 3). The helical twist was generally a few degrees above 30° and similar to that for the normal G:C duplex. There was a general reduction in inclination for the G^S^-substituted duplex compared to the normal one, and this was sometimes much larger than the 3° reduction observed in reference [15]. The structure of the G^S^:C duplex was thus overall similar to that of the G:C duplex with the exception of the opening. The structural differences noted for G:T compared to G:C do not apply to G^S^:C versus G:C, leaving them as possible candidates for recognition. The frequency of hydration of S6 in the G^S^:C duplex is however far lower than that of O6 in the G:T duplex providing evidence against the role of hydration in mismatch recognition.

### G^S^:T duplex

**Fig 9:**
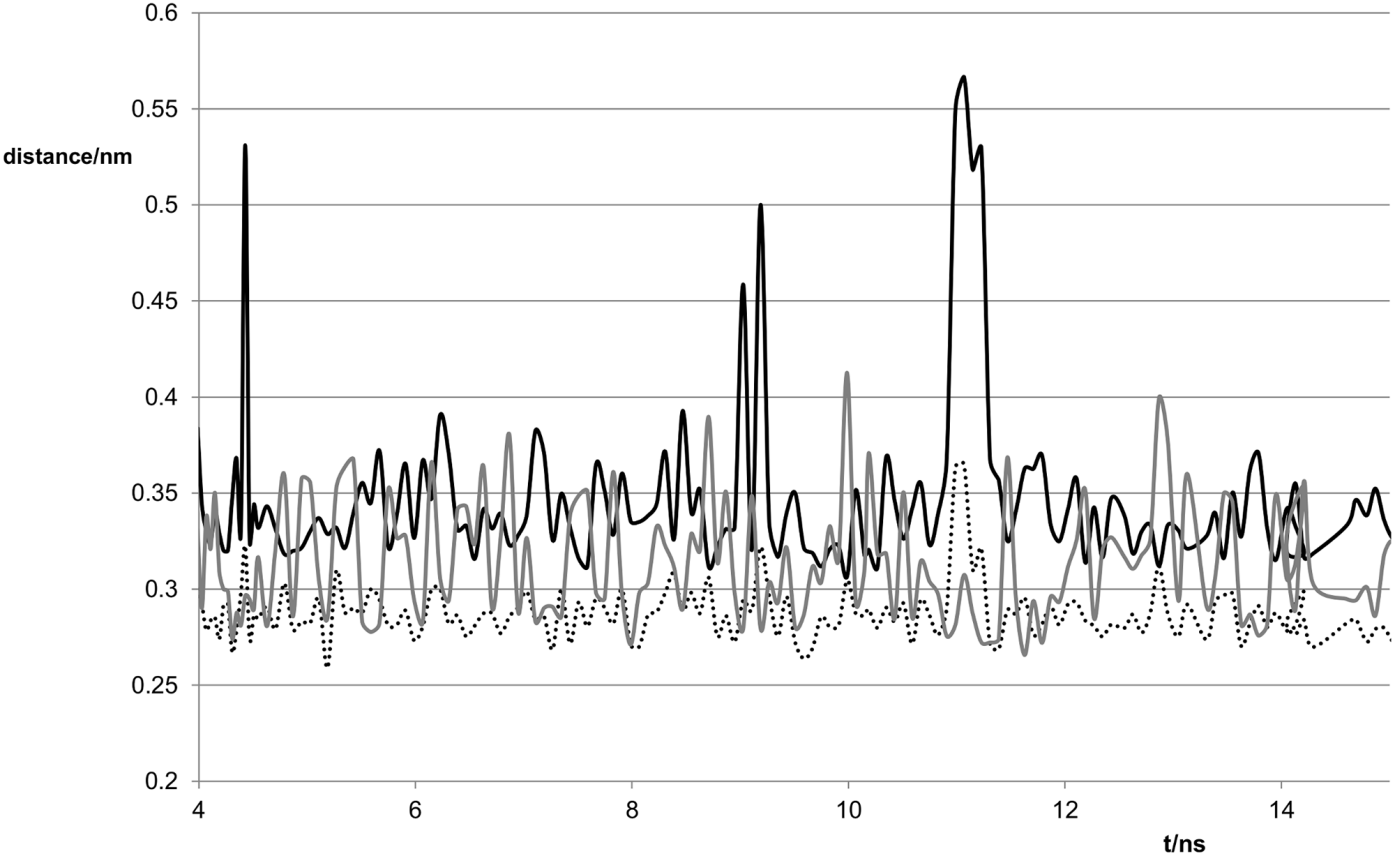
Variation of distances between potentially hydrogen-bonded atoms with time for the G^S^:T duplex. Black line S6-N3 distance, grey line N2-O2 distance, dotted line N1–O2 distance.

The N1-O2 hydrogen bond remained throughout the simulation and the N1-O2 distance was similar to that in G:T. The N2-O2 distance (cf: Fig 3c) was generally longer than the N1-O2 distance and was more variable, frequently reaching 0.35 or even >4 nm (Fig 9).

The S6-N3 distance was generally longer than either of the N-O distances as would be expected for the weakly hydrogen-bonding sulfur, but in some parts of the trajectory (for example around 4, 9 and 11 ns) this distance was far too long for any hydrogen-bonding to occur. Twist, shear and roll values at the mismatch site resembled those of the G:T mismatch, see Table 3. The opening was much larger even than for the G^S^:C duplex and displayed a greater variation (see Fig 8). The maxima coincide with the larger S6-N3 distances and are probably a consequence of the base pair being bonded through only one hydrogn bond. The base-pair buckle was close to 0° rather than the -11° observed previously [14]. As for the G:T mismatch, the value of the twist parameter had a larger standard deviation and was much smaller than the mean for AG^S^/TT and larger than the mean for G^S^G/CT. The mean for G^S^G/CT was however larger than for GG/CT.

The major grooves had similar values for both G^S^:T and G^S^:C duplexes. For G^S^:C the variation in the major groove was similar to that for G:C The minor groove for G^S^:T was smaller than those for the other three duplexes studied.

The number of waters in the first and second shell for the G^S^ was similar to that of the other duplexes, 48 and 106 respectively. Looking at hydrogen-bonding of S in G^S^ to water, we found that this occurred 4.44% of the time for the G^S^:T mismatch. This is less than for G:C but considerably greater than for G^S^:C and similar to that for G:T. The similarity between the twist for G:T and G^S^:T argues for this as a possible feature for recognition.

The very large increase in opening from G:T to G^S^:T (see Fig 8) and the increased variability of this parameter is the most striking difference between this oligomer and the other three studied. This may make it possible for the mismatch recognition protein to attach to G^S^. This could lend suport to an alternative model for apoptosis [6, 7] in which MSH2/MSH6 and MLH1/PMS2 bind to the damaged base and activate downstream DNA damage-response pathways. The large opening in G^S^:T may also contribute to the role of thioguanine in DNA acting to generate reactive oxygen species [31].

## Conclusions

Four duplexes based on d(5’-GCTAA**G**GAAAGCC-3’) were studied. We considered differences between the duplexes that might lead the repair mechanism to recognise G:T mismatches and whether these differences were also present in G^S^:T mismatches. Comparison with previous work [11] indicates that the average structure rather than the variability of the structure informs recognition of G:T

The main distinguishing feature of the structure of the G:T duplex that we found is a smaller twist in the adjacent base-pair step. A consistent difference in the shear value was also noted. The G^S^:T duplex resembled the G:T duplex in its twist and roll values. The twist and shear values differentiated mismatch oligomers, G:T and G^S^:T from their equivalent oligomers without the mismatch G:C and G^S^:C. In particular the twist value for both these duplexes was considerably greater than that for the G:C and G^S^:C duplexes and this may be a marker for recognition by the mismatch system and the reason that G^S^:T is only repaired to G^S^:C.

However we note that the value of the opening for the G^S^:T duplex is far larger than for the other three duplexes. We suggest that this feature may lead to attachment of the mismatch protein to G^S^ and to availabilty of S6 in the G^S^:T duplex to reactive oxygen species and hence to oxidation. Both of these would lead to apoptosis rather than mismatch repair.

We therefore conclude that shear and twist values are recognised by the repair protein leading to replacement of T by C in both the normal and thio-substituted oligomer, but that the very large and variable opening of G^S^:T leads to an increased chance of oxidation of the sulfur and finally to cellular apoptosis.

## Supporting information

S1 Fig Mass-weighted rms for the G:C duplex as a function of time. (TIF)

S2 Fig Mass-weighted rms for the G:T duplex as a function of time. (TIF)

S3 Fig Mass-weighted rms for the G^S^:C duplex as a function of time. (TIF)

S4 Fig Mass-weighted rms for the G^S^:T duplex as a function of time. (TIF)

S5 Fig Average structure of G^S^:T duplex over 8–9 ns. Atoms in bases G6 and T21 are shown explicitly. Yellow ball = sulphur.
(TIF)

S6 Fig Average structure of G^S^:T duplex over 10–11 ns. Atoms in bases G6 and T21 are shown explicitly. Yellow ball = sulphur.
(TIF)

S7 Fig Average structure of G^S^:T duplex over 13–14 ns. Atoms in bases G6 and T21 are shown explicitly. Yellow ball = sulphur.
(TIF)

S8 Fig Average structure of most visited cluster of G:C duplex. (TIF)

S9 Fig Average structure of most visited cluster of G:T duplex. (TIF)

S10 Fig Average structure of most visited cluster of G^S^:C duplex. (TIF)

S11 Fig Average structure of most visited cluster of G^S^:T duplex. (TIF)

S1 Text Structural parameters from X3DNA of structures averaged over nanosecond intervals from 3–15 ns for all 4 duplexes. Tables A–D.
(DOCX)

S1 Table Atomic charges for thioguanosine. Only charges differing from those in reference [15] are given.
(DOCX)
