# Supplementary figures and images for: The effect of S-substitution at the O6-guanine site on the structure and dynamics of a DNA oligomer containing a G:T mismatch
Source: PLoS One. 2017 Sep 14;12(9):e0184801. doi: 10.1371/journal.pone.0184801 (PMC5599020; doi:10.1371/journal.pone.0184801)

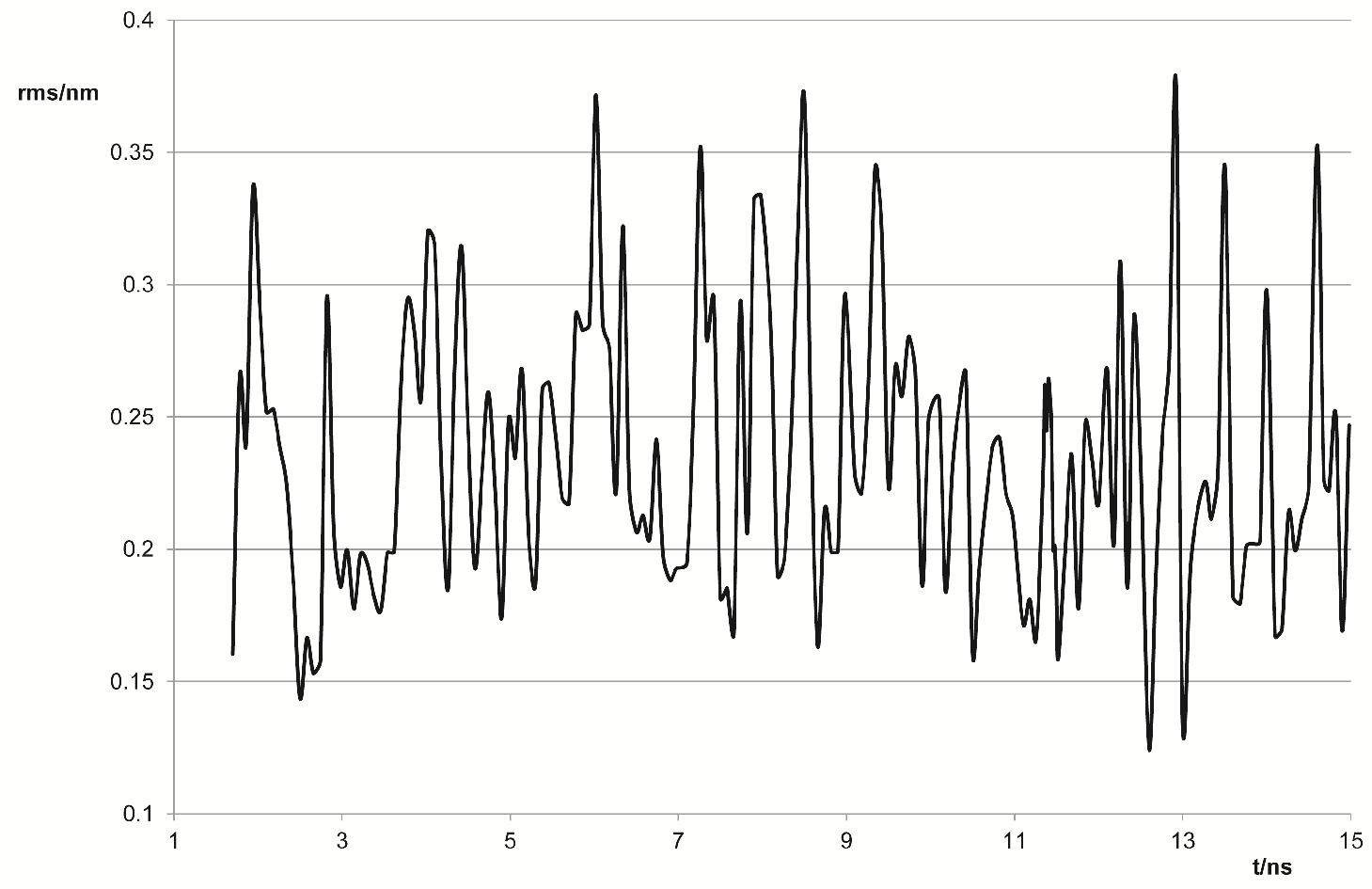

Supplement: S1 Fig — (TIF) [file pone.0184801.s001.tif]

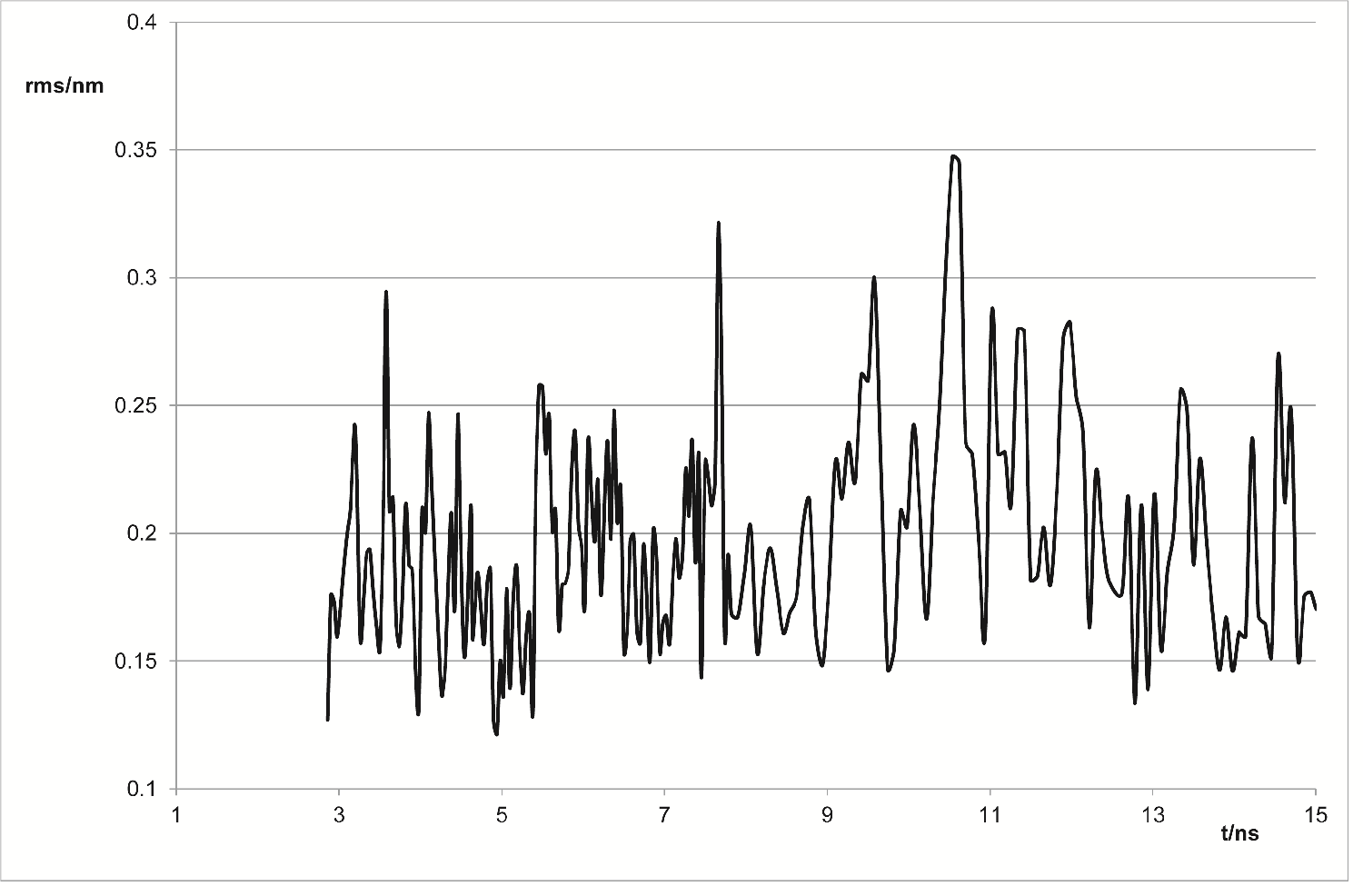

Supplement: S2 Fig — (TIF) [file pone.0184801.s002.tif]

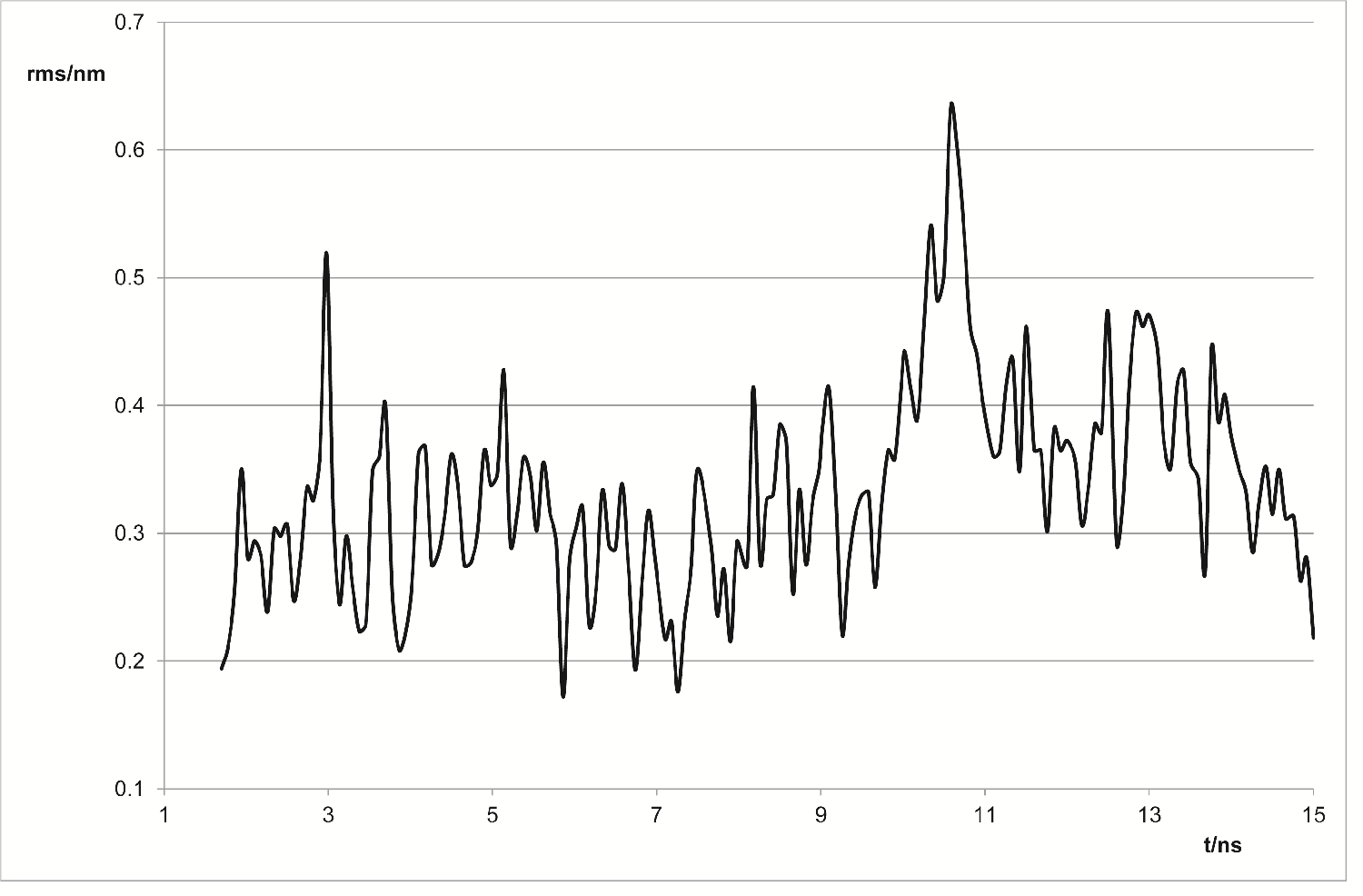

Supplement: S3 Fig — (TIF) [file pone.0184801.s003.tif]

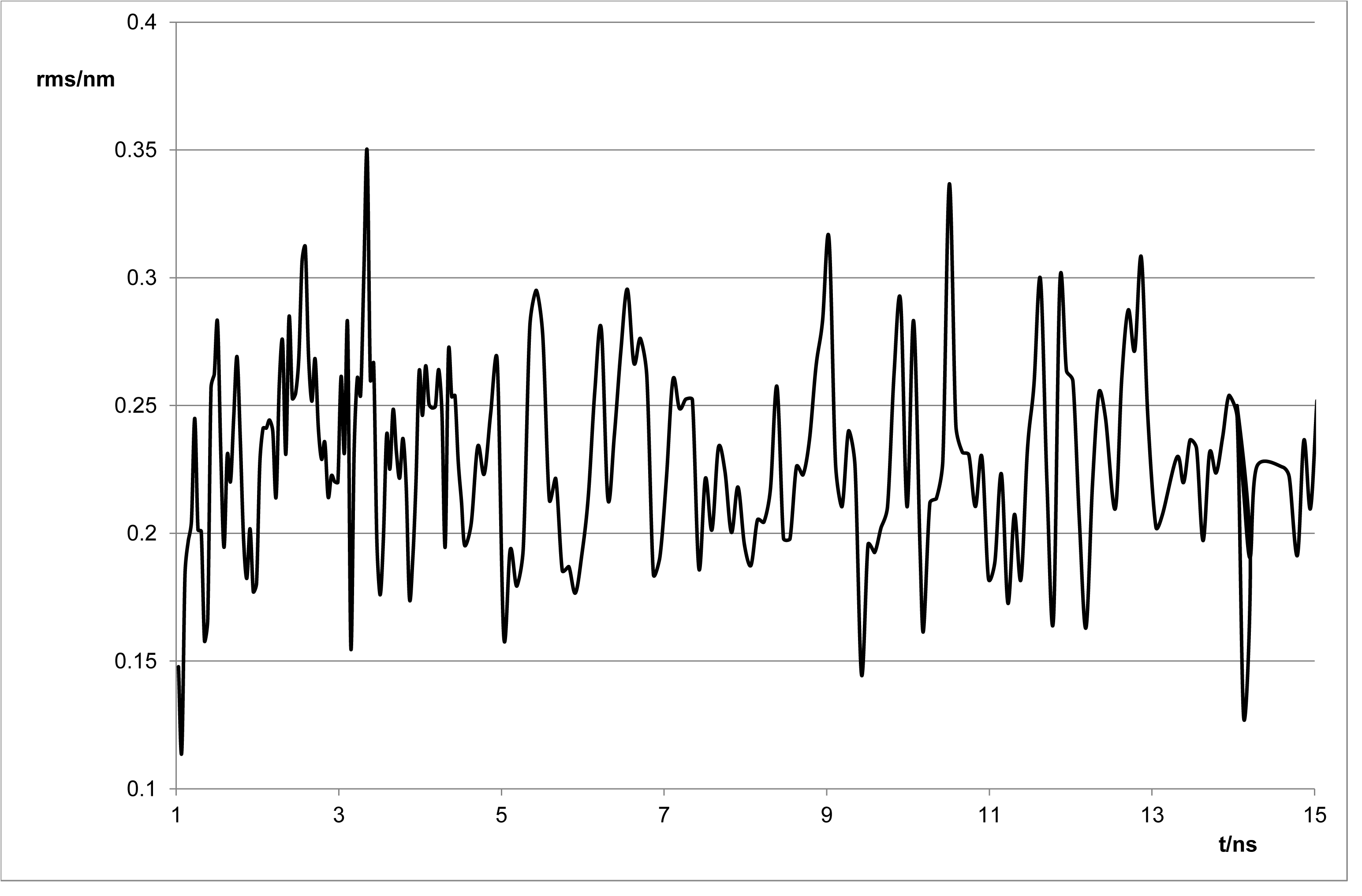

Supplement: S4 Fig — (TIF) [file pone.0184801.s004.tif]

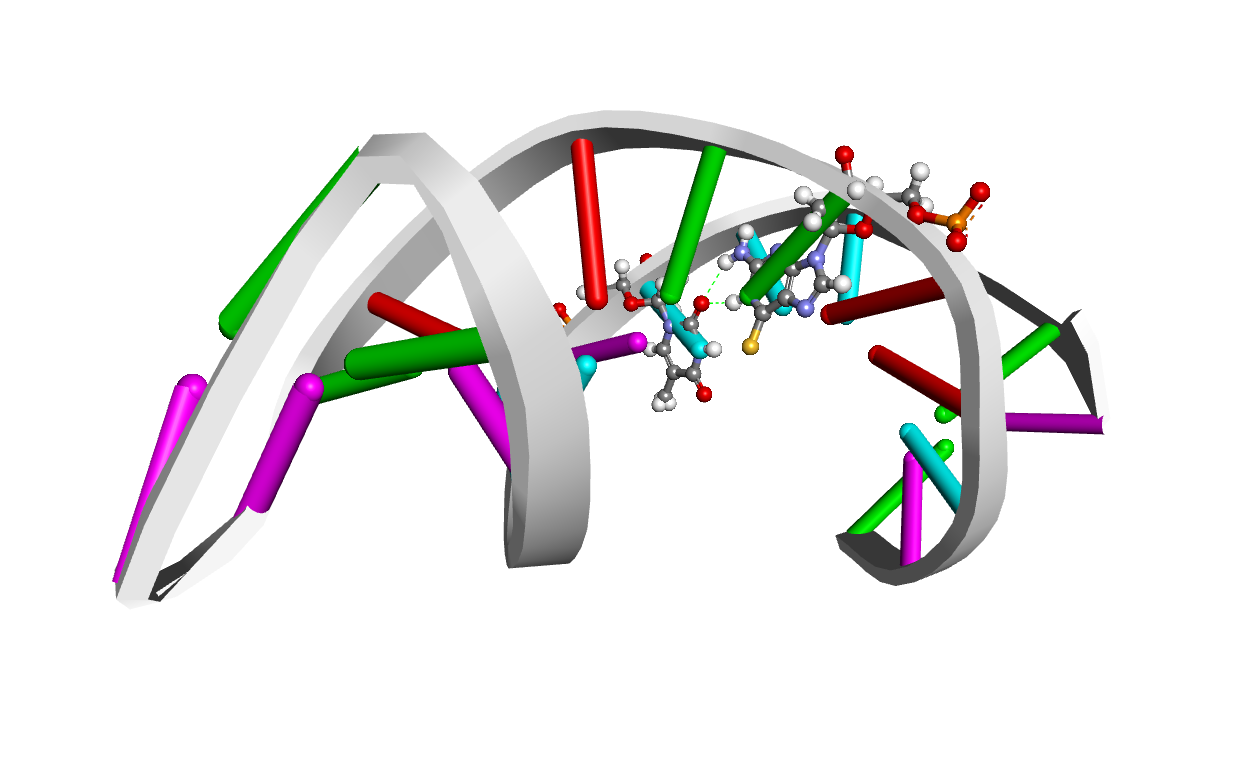

Supplement: S5 Fig — Atoms in bases G6 and T21 are shown explicitly. Yellow ball = sulphur. (TIF) [file pone.0184801.s005.tif]

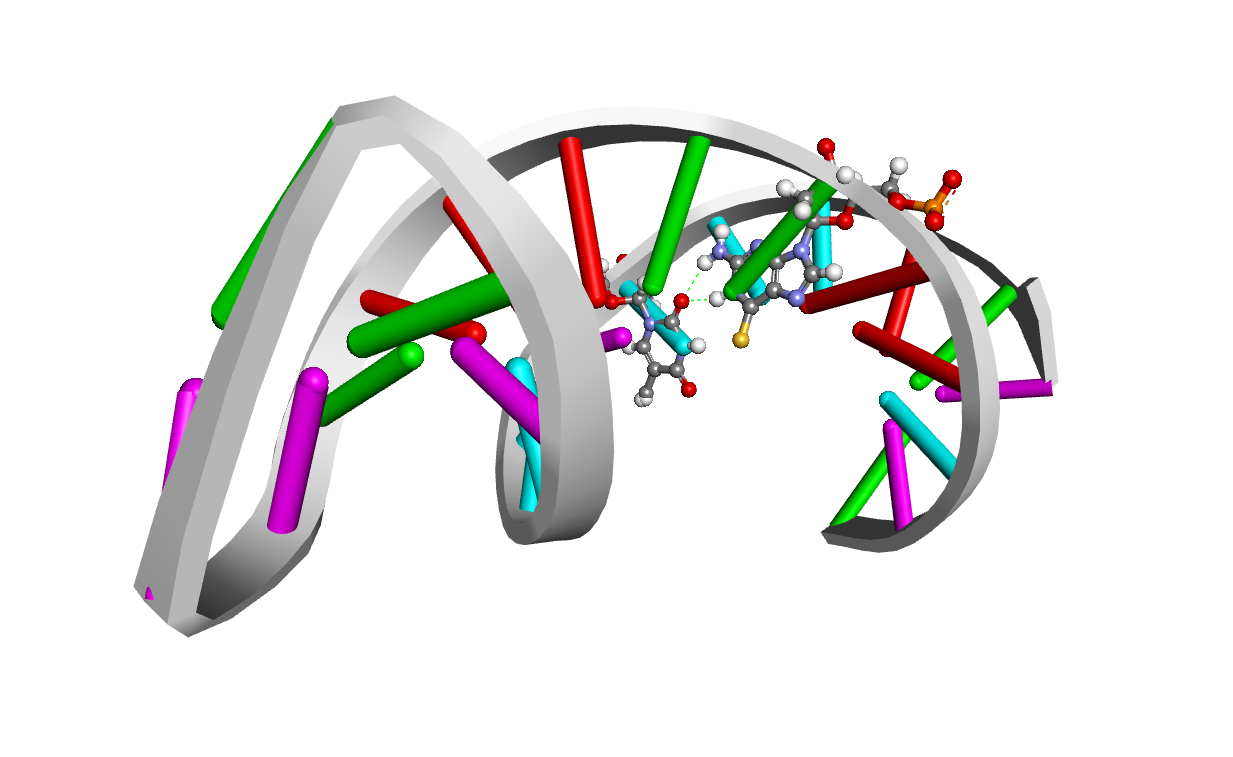

Supplement: S6 Fig — Atoms in bases G6 and T21 are shown explicitly. Yellow ball = sulphur. (TIF) [file pone.0184801.s006.tif]

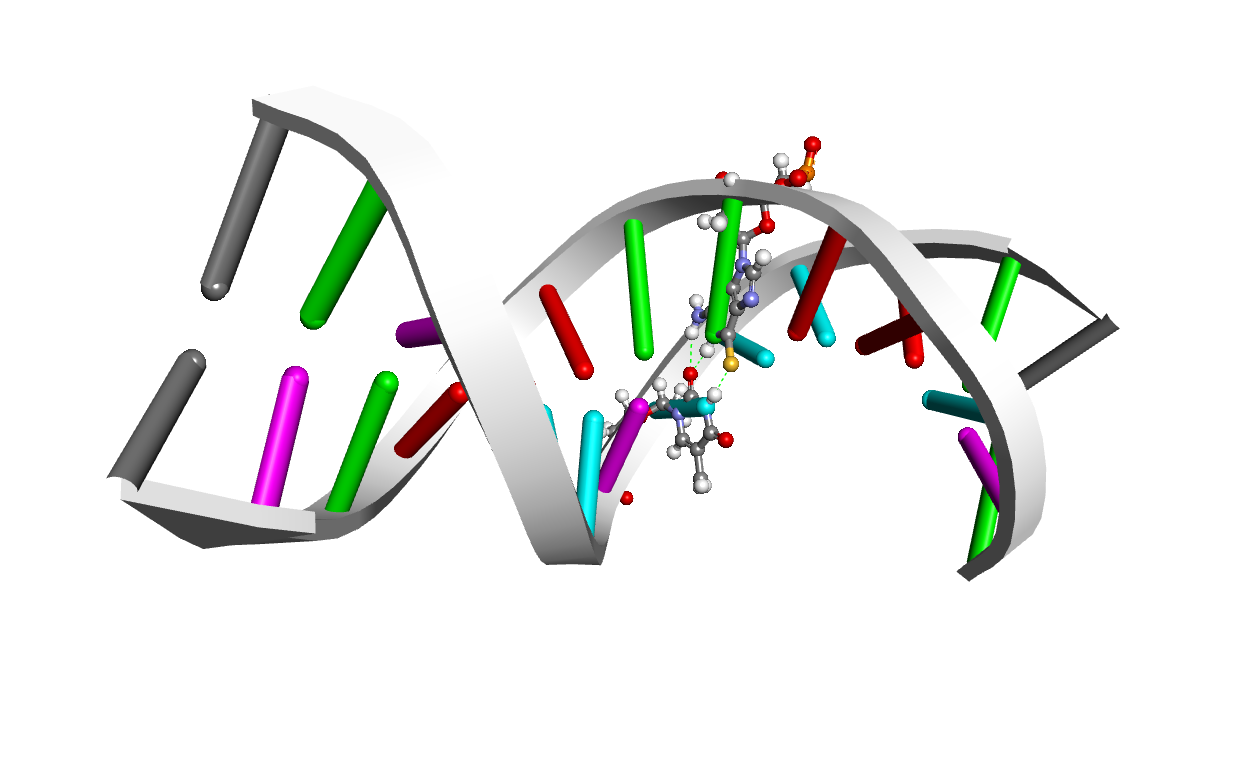

Supplement: S7 Fig — Atoms in bases G6 and T21 are shown explicitly. Yellow ball = sulphur. (TIF) [file pone.0184801.s007.tif]

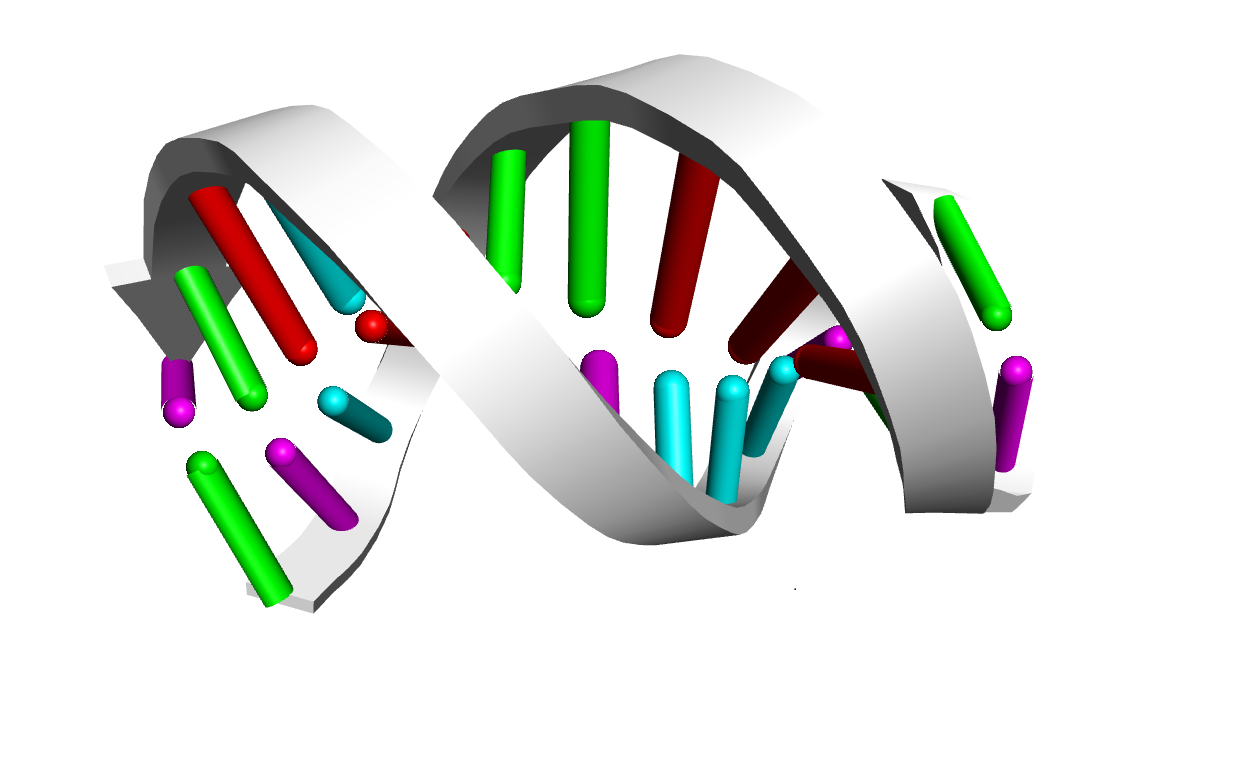

Supplement: S8 Fig — (TIF) [file pone.0184801.s008.tif]

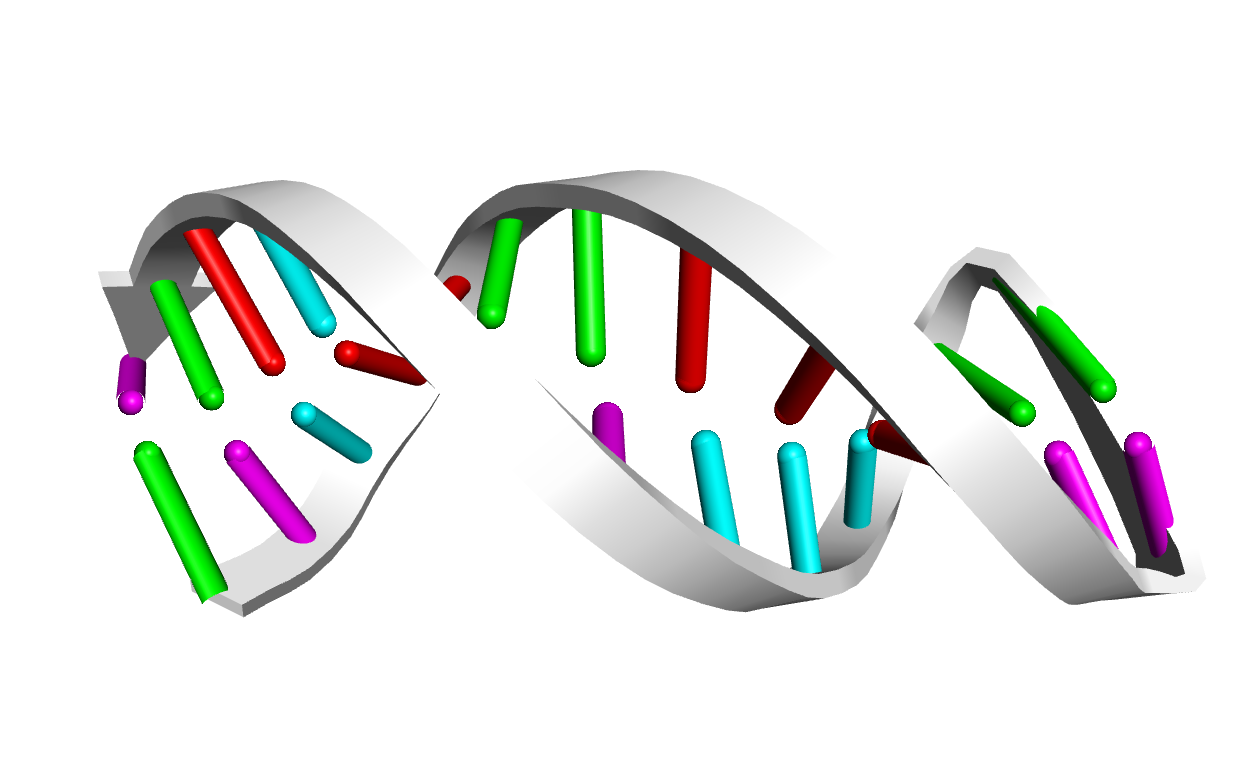

Supplement: S9 Fig — (TIF) [file pone.0184801.s009.tif]

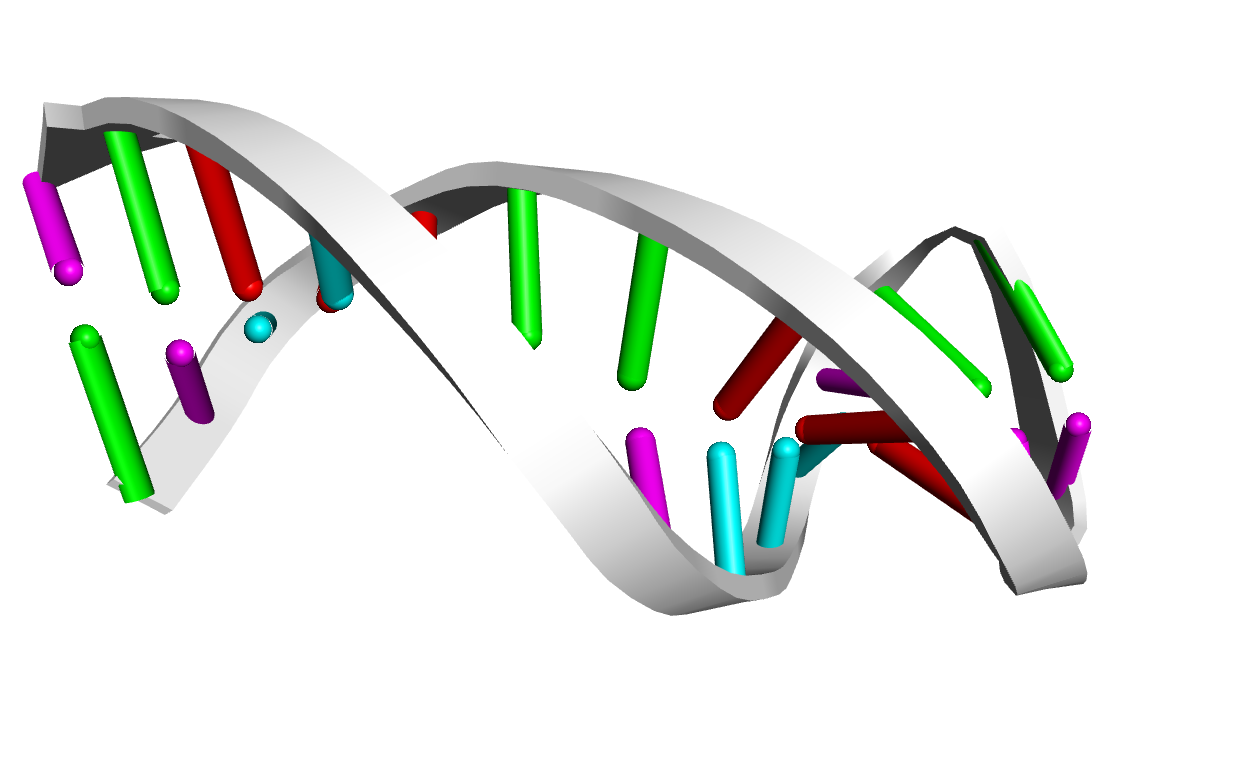

Supplement: S10 Fig — (TIF) [file pone.0184801.s010.tif]

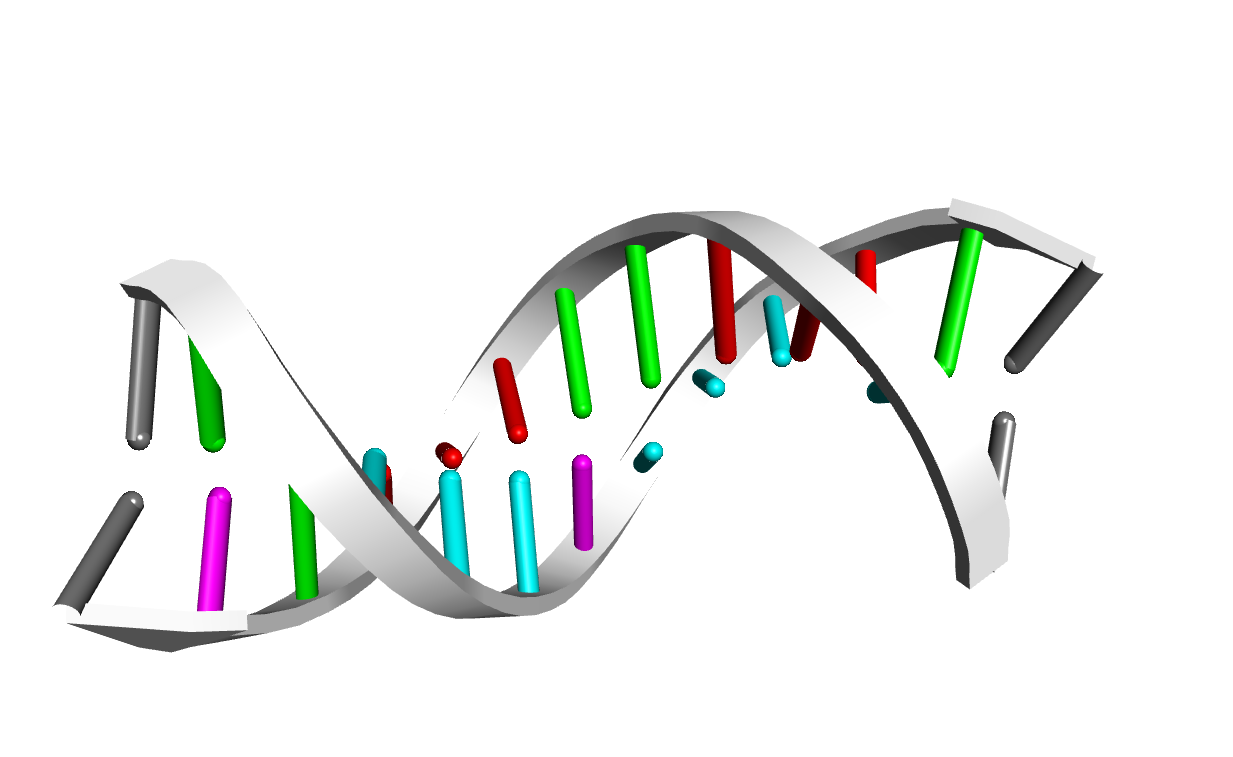

Supplement: S11 Fig — (TIF) [file pone.0184801.s011.tif]
