# Supplementary material for: The effect of S-substitution at the O6-guanine site on the structure and dynamics of a DNA oligomer containing a G:T mismatch
Source: PLoS One. 2017 Sep 14;12(9):e0184801. doi: 10.1371/journal.pone.0184801 (PMC5599020; doi:10.1371/journal.pone.0184801)
Supplement: S1 Text — Tables A–D. (DOCX) [file pone.0184801.s012.docx]

Supplementary tables

Table A Structural parameters for the averaged structures for individual nanoseconds of the G:C duplex

| t/ns | shear/nm | stretch/nm | opening/° | helical twist/° | major groove/nm | minor groove/nm | rmsd G6/nm | rmsd C21/nm |
| --- | --- | --- | --- | --- | --- | --- | --- | --- |
| 4 | -0.026 | -0.027 | -1.08 | 28.55 | 2.21 | 1.21 | 0.0042 | 0.0035 |
| 5 | -0.014 | -0.024 | -0.29 | 31.86 | 2.09 | 1.19 | 0.0069 | 0.0039 |
| 6 | 0.001 | -0.048 | -1.51 | 29.29 | 2.1 | 1.27 | 0.0089 | 0.0077 |
| 7 | -0.014 | -0.023 | -2.51 | 30.85 | 1.98 | 1.28 | 0.004 | 0.0029 |
| 8 | -0.104 | -0.021 | -0.56 | 30.9 | 2.03 | 1.25 | 0.0041 | 0.0046 |
| 9 | -0.022 | -0.025 | 0.14 | 31.3 | 2.2 | 1.2 | 0.0037 | 0.0048 |
| 10 | -0.028 | -0.023 | 1.2 | 35.48 | 1.82 | 1.11 | 0.0048 | 0.003 |
| 11 | -0.019 | -0.024 | 1.46 | 31.5 | 2.03 | 1.25 | 0.0054 | 0.0043 |
| 12 | -0.016 | -0.014 | -0.28 | 30.73 | 2.13 | 1.26 | 0.0038 | 0.0027 |
| 13 | -0.104 | -0.04 | -0.57 | 26.81 | 2.22 | 1.11 | 0.01 | 0.0047 |
| 14 | -0.008 | -0.016 | -0.76 | 30.65 | 1.99 | 1.21 | 0.0036 | 0.003 |
| 15 | -0.01 | -0.101 | 0.19 | 30.93 | 2.21 | 1.28 | 0.0043 | 0.0039 |

Table B Structural parameters for the averaged structures for individual nanoseconds of the G:Tduplex

| t/ns | shear/nm | stretch/nm | opening/° | helical twist/° | major groove/nm | minor groove/nm | rmsd G6/nm | rmsd T21/nm |
| --- | --- | --- | --- | --- | --- | --- | --- | --- |
| 4 | -0.216 | -0.087 | 0.43 | 38.96 | 1.98 | 1.07 | 0.0106 | 0.008 |
| 5 | -0.215 | -0.064 | 4.39 | 38.09 | 1.97 | 1.18 | 0.0095 | 0.0086 |
| 6 | -0.219 | -0.046 | 0.92 | 37.99 | 2.01 | 1.13 | 0.0046 | 0.0038 |
| 7 | -0.215 | -0.052 | 1.64 | 39.3 | 1.97 | 1.18 | 0.005 | 0.0037 |
| 8 | -0.225 | -0.054 | 1.1 | 38.44 | 1.99 | 1.24 | 0.005 | 0.0038 |
| 9 | -0.228 | -0.076 | -1.78 | 39.24 | 1.92 | 1.18 | 0.0071 | 0.0043 |
| 10 | -0.216 | -0.063 | 1.2 | 38.35 | 2.01 | 1.06 | 0.006 | 0.0059 |
| 11 | -0.232 | -0.054 | 1.59 | 42 | 2.05 | 1.11 | 0.0036 | 0.0026 |
| 12 | -0.222 | -0.054 | 1.08 | 37.87 | 1.97 | 1.25 | 0.0038 | 0.0041 |
| 13 | -0.234 | -0.056 | -1.21 | 40.18 | 1.91 | 1.22 | 0.0034 | 0.0032 |
| 14 | -0.221 | -0.048 | 1.28 | 37.92 | 2.04 | 1.17 | 0.0028 | 0.0038 |
| 15 | -0.239 | -0.052 | 1.64 | 40.48 | 2.21 | 1.09 | 0.0037 | 0.0029 |

Table C Structural parameters for the averaged structures for individual nanoseconds of the G^S^:Cduplex

| t/ns | shear/nm | stretch/nm | opening/° | helical twist/° | major groove/nm | minor groove/nm | rmsd G^S^6/nm | rmsd C21/nm |
| --- | --- | --- | --- | --- | --- | --- | --- | --- |
| 4 | -0.031 | 0.003 | 8.99 | 29.82 | 2.25 | 1.23 | 0.0049 | 0.0037 |
| 5 | 0.006 | 0.003 | 6.52 | 33.59 | 1.77 | 1.24 | 0.0049 | 0.0055 |
| 6 | -0.017 | 0.006 | 7.45 | 30.69 | 2.18 | 1.25 | 0.0038 | 0.0029 |
| 7 | -0.031 | -0.018 | 7.90 | 32.39 | 2.29 | 1.24 | 0.0032 | 0.0037 |
| 8 | 0.002 | 0.006 | 7.45 | 27.59 | 2.37 | 1.26 | 0.0035 | 0.005 |
| 9 | -0.012 | 0.006 | 7.65 | 29.41 | 1.88 | 1.04 | 0.0045 | 0.0099 |
| 10 | 0.001 | -0.003 | 7.29 | 26.7 | 2.15 | 1.21 | 0.0064 | 0.0049 |
| 11 | -0.012 | 0.006 | 7.65 | 29.41 | 1.88 | 1.04 | 0.045 | 0.0077 |
| 12 | 0.005 | 0.003 | 7.25 | 35.15 | 2.05 | 1.15 | 0.0031 | 0.0036 |
| 13 | -0.005 | 0.003 | 5.58 | 30.15 | 2.25 | 1.23 | 0.0044 | 0.0036 |
| 14 | -0.024 | -0.012 | 8.88 | 32.02 | 2.23 | 1.12 | 0.0087 | 0.0065 |
| 15 | -0.021 | -0.034 | 6.28 | 31.74 | 2.21 | 1.13 | 0.0079 | 0.0060 |

Table D Structural parameters for the averaged structures for individual nanoseconds of the G^S^:Tduplex

| t/ns | shear/nm | stretch/nm | opening/° | helical twist/° | major groove/nm | minor groove/nm | rmsd G^S^6/nm | rmsd T21/nm |
| --- | --- | --- | --- | --- | --- | --- | --- | --- |
| 4 | -0.216 | 0.021 | 20.32 | 40.48 | 2.07 | 1.12 | 0.041 | 0.046 |
| 5 | -0,21 | -0.005 | 15.63 | 37.17 | 2.24 | 1.16 | 0.052 | 0.042 |
| 6 | -0.214 | -0.003 | 15.19 | 37.39 | 1.99 | 1.11 | 0.065 | 0.028 |
| 7 | -0.224 | -0.04 | 14.43 | 38.44 | 2.06 | 1.29 | 0.029 | 0.046 |
| 8 | -0.211 | 0.002 | 15.42 | 39.36 | 2.15 | 1.13 | 0.022 | 0.037 |
| 9 | -0.22 | 0.016 | 17.65 | 37.01 | 2.01 | 1.21 | 0.043 | 0.041 |
| 10 | -0.212 | -0.01 | 12.78 | 38.66 | 2.14 | 1.1 | 0.029 | 0.036 |
| 11 | -0.219 | 0.031 | 21.56 | 38.11 | 2.13 | 1.18 | 0.048 | 0.04 |
| 12 | -0.223 | 0.305 | 25.51 | 40.84 | 2.02 | 1.1 | 0.037 | 0.035 |
| 13 | -0.022 | -0.008 | 13.24 | 40.60 | 1.92 | 1.06 | 0.0038 | 0.0033 |
| 14 | -0.223 | -0.011 | 14.63 | 39.79 | 1.92 | 1.03 | 0.058 | 0.035 |
| 15 | -0.217 | -0.007 | 16.14 | 40.22 | 2.14 | 1.10 | 0.0026 | 0.0044 |
