# Supplementary material for: The effect of S-substitution at the O6-guanine site on the structure and dynamics of a DNA oligomer containing a G:T mismatch
Source: PLoS One. 2017 Sep 14;12(9):e0184801. doi: 10.1371/journal.pone.0184801 (PMC5599020; doi:10.1371/journal.pone.0184801)
Supplement: S1 Table — Only charges differing from those in reference [15] are given. (DOCX) [file pone.0184801.s013.docx]

# Table S1

Table S1 Atomic charges for thioguanosine. Only charges differing from those in reference 15 are given.

| **atom** | **charge** |
| --- | --- |
| C1’ | +0.4312 |
| H1’ | -0.0564 |
| N9 | -0.2781 |
| C8 | +0.3883 |
| H8 | +0.1579 |
| N7 | -0.7585 |
| C5 | +0.4353 |
| C6 | -0.3799 |
| S6 | -0.3159 |
| N1 | -0.0349 |
| H1 | +0.5337 |
| C2 | +0.6964 |
| N2 | -1.0912 |
| H21 | +0.4788 |
| H22 | +0.4788 |
| N3 | -0.6549 |
| C4 | +0.3838 |
